# Basal Differences in the Transcriptional Profiles of Tomato Roots Associated with the Presence/Absence of the Resistance Gene *Mi-1* and Time-Course Changes During the Compatible and Incompatible Interactions with the Root-Knot Nematode *Meloidogyne javanica*

**DOI:** 10.3390/plants15101428

**Published:** 2026-05-07

**Authors:** Ana Rico, Alicia Ávila, Mariana Emiliozzi, Irene López-Vidriero, José M. Franco-Zorrilla, Gloria Nombela

**Affiliations:** 1 Department of Plant Protection, Institute for Agricultural Sciences (ICA), Spanish National Research Council (CSIC), Serrano 115 Dpdo., 28006 Madrid, Spain; ana.ricosanz@gmail.com (A.R.); avilop.alicia@gmail.com (A.Á.); marianaemiliozzi@gmail.com (M.E.); 2 Genomics Unit, Centro Nacional de Biotecnología (CNB), Spanish National Research Council (CSIC), Darwin 3, 28049 Madrid, Spain; irene.lopez@aei.gob.es (I.L.-V.); jmfranco@cnb.csic.es (J.M.F.-Z.)

**Keywords:** host-plant resistance, microarrays, *Meloidogyne javanica*, *Mi-1* gene, root-knot nematodes, tomato

## Abstract

The *Mi-1* gene of tomato is responsible for the resistance of certain genotypes to root-knot nematodes or RKN (*Meloidogyne* spp.) and other harmful organisms such as aphids or whiteflies, in a complex cascade of transcriptional changes in which other tomato genes are also involved. The objective of this study was to gain a deeper understanding of the *Mi-1*-mediated resistance of tomato to *Meloidogyne javanica* using oligonucleotide microarrays to identify additional plant genes involved in the compatible or incompatible tomato/nematode interactions. Microarray analysis was selected as it has been widely used to identify genes involved in plant resistance to pests and pathogens. In a first phase of the present work, the roots of uninfested tomato plants were analyzed, comparing the transcriptional profiles of susceptible (Moneymaker) and resistant (Motelle) cultivars. In Motelle, 180 transcripts were more expressed than in Moneymaker and only 44 transcripts showed lower expression. Motelle showed higher activity in salicylic, jasmonic and ethylene pathways, while the GAI protein was strongly repressed compared to Moneymaker. These and other basal differences provided valuable information on candidate genes associated with the presence of the *Mi-1* gene in Motelle. Subsequent infection by *M. javanica* triggered an intense transcriptional reprograming that increased over time throughout both compatible (Moneymaker) and incompatible (Motelle) interactions, with scarce genes common to both interactions. At the early phase of infection (2 dpi), genes for the cell wall, hormones, RNA, stress, and transport were up-regulated in the compatible interaction, and signaling, protein, and redox genes were down-regulated; in the incompatible interaction, protease inhibitors were up-regulated, and hormone and RNA genes were down-regulated. Later (12 dpi), genes for hormones, the cell wall, RNA, stress, defense, and development were up-regulated in the compatible interaction, while transport and some stress/defense genes were down-regulated; the incompatible interaction showed mixed regulation within hormone, stress, and defense genes.

## 1. Introduction

Tomato (*Solanum lycopersicum*) is the most common vegetable in the world and one of the plants with the largest economic value, with a global production of more than 188 million tons (FAOSTAT, 2024). Moreover, it is a perennial annual angiosperm having several advantages as a model plant, including a small genome, a short generation time, the availability of transformation protocols, and genetic and genomic resources [1], all of which have led to the complete sequencing of tomato genome [2,3]. Root-knot nematodes or RKN (*Meloidogyne* spp.) constitute the main phytonematological problem of tomato due to their rapid expansion, high frequency of infection, and ability to reduce yield [4]. *Meloidogyne* spp. are sedentary endoparasites of a polyphagous nature and some species of this genus are considered to be of the most pathogenic importance worldwide. They can infect thousands of plant species, including extensive, horticultural and fruit crops, seriously affecting production and causing huge economic losses [5]. The nematode life cycle begins in the soil, where infective juveniles (J2) hatch, penetrate the root near the apex, and migrate between the intercellular spaces of the cortical parenchyma to reach the vascular cylinder. The secretion of cell wall-modifying enzymes from the nematode stylet induces the transformation of several cells into giant cells, leading to the feeding site and resulting in thickened root galls in the susceptible plant [6]. However, resistant tomato varieties exist where J2 are able to penetrate the roots and migrate to initiate feeding near the vascular element, but, at this stage, an active plant defense based on a hypersensitive response (HR) is triggered in the area near the head of the juvenile, inhibiting nematode feeding and subsequent development [7,8]. This resistance is mediated by the *Mi-1* gene, introduced into cultivated tomato from its wild relative, *S. peruvianum* [9]. This gene confers resistance to three species of root-knot nematodes (*M. arenaria*, *M. javanica* and *M. incognita*) [10], as well as to virus-transmitter insects such as the potato aphid *Macrosiphum euphorbiae* [11], the sweetpotato whitefly *Bemisia tabaci* [12] and the tomato psyllid *Bactericerca cockerelli* [13].

The *Mi-1* gene was localized in a 52 Kb region of the short arm of chromosome 6 of tomato and subsequently cloned [14,15]. This gene codifies for MI-1, a CC-NB-LRR protein with 1257 aminoacids, similar to other R proteins [15,16,17]. The *Mi-1* gene is constitutively present in every tissue of resistant tomato very early in development [18], and the MI-1 protein remains inactive while not necessary. Upon detection of effector molecules that indicate the presence of the nematode, this protein experiences a conformational change and activates different signals leading to the resistance response [19].

Plant resistance is the result of the agreed efforts of different genes in certain signal transduction pathways leading to defense against the attacker organism, and the presence and function of all these genes are necessary for the resistance to work. So, additional genes have been identified in tomato that are necessary for the *Mi-1*-mediated resistance, such as *Rme1* against aphids, nematodes and whiteflies [18,20,21] and *Hsp90* and *Sgt1* against nematodes and aphids [22]. A model of interaction between the proteins encoded by these genes has been proposed: MI-1, HSP90 and SGT1 form a R-signaling complex that guards RME1 [22]. Since then, some strains of *M. javanica* virulent to carrying-*Mi* plants have been detected [23,24,25]. Although a lot of research groups continued working on plant–nematode interaction [26,27,28], more research on molecular aspects of plant resistance is necessary, particularly on the mechanisms regulating processes related to resistance to nematodes and to identifying new genes that control and modulate the resistance response.

High-performance technologies such as microarrays are among the key tools in functional genomic analysis for their ability to monitor a large number of genes simultaneously and to identify alterations in their expression [29]. In the case of plants, the increased number and quality of available sequence databases has allowed the study of transcriptional reprogramming in many different physiological situations [30,31]. This includes changes in plant response to insect feeding [32,33,34,35,36,37,38] or to the infection with bacterial pathogens [38,39] or plant–pathogenic nematodes [40,41,42,43]. More specifically, transcriptome studies reveal complex gene expression and metabolic networks that took place in plant–RKN interaction. Most research groups have used microarray analysis to identify transcriptomic changes due to RKN feeding during compatible interactions with Arabidopsis [44,45], tomato [46,47], soybean [48], common bean [49] and potato [50], or to compare tomato and Arabidopsis during development of giant cells [43].

However, studies using microarray to analyze the mechanisms that regulate in tomato the *Mi-1*-gene resistance to this type of nematodes have been scarce. Schaff [51] constructed a tomato cDNA microarray with 1547 selected root-expressed genes and found that, in the absence of RKN, only one gene encoding a glycosyltransferase was differentially regulated between the transcriptomes of resistant and susceptible cultivars. They used the same partial microarray to analyze both compatible and incompatible interactions in 3- to 4-week-old seedlings inoculated with *M. hapla* or *M. incognita*. An Affymetrix Arabidopsis ATH1 genome array GeneChip was used to demonstrate the contribution of WRKY72-type transcription factors to basal immunity in tomato and Arabidopsis as well as the *Mi-1*-mediated resistance of tomato seedlings to *M. incognita* [52]. A more recent analysis focused on tomato/*M. incognita* interaction, comparing susceptible and resistant responses in 5-week-old plants [53]. Closely related to the work presented here was the study by our research group in which, with a similar approach, microarrays were used in tomato leaf tissues to detect differences between the transcriptional profiles of plants carrying and those lacking the *Mi-1* gene, and how such differences are modified after infestation by the whitefly *B. tabaci* [38].

In the present work, the GeneChip^®^ Tomato Genome Array (Affymetrix^®^) has been used in an unbiased study to compare, for the first time, global expression changes of over 9200 transcripts in the root system of adult (8-week-old) tomato plants in the presence or absence of the R gene *Mi-1*. In the first phase of this study, uninfested root tissues of tomato plants of a susceptible cultivar (Moneymaker) and a *Mi-1*-bearing (resistant) cultivar (Motelle) were analyzed and their transcriptional profiles were compared to detect basal differences associated with the presence/absence of the *Mi-1* gene. In a second phase of the study, roots of each tomato cultivar were infected with *M. javanica* and then compared by microarray to uninfected roots of the same cultivar to analyze both the compatible (Moneymaker) and incompatible (Motelle) interactions. To better understand these changes and why resistant tomato is successful in preventing infection, two time points were considered: an early stage at 2 days post-inoculation (2 dpi) and another later stage at 12 days post-inoculation (12 dpi).

## 2. Results

### 2.1. Basal Differences Between Moneymaker and Motelle in the Absence of Nematode Infection

**Figure 1:**
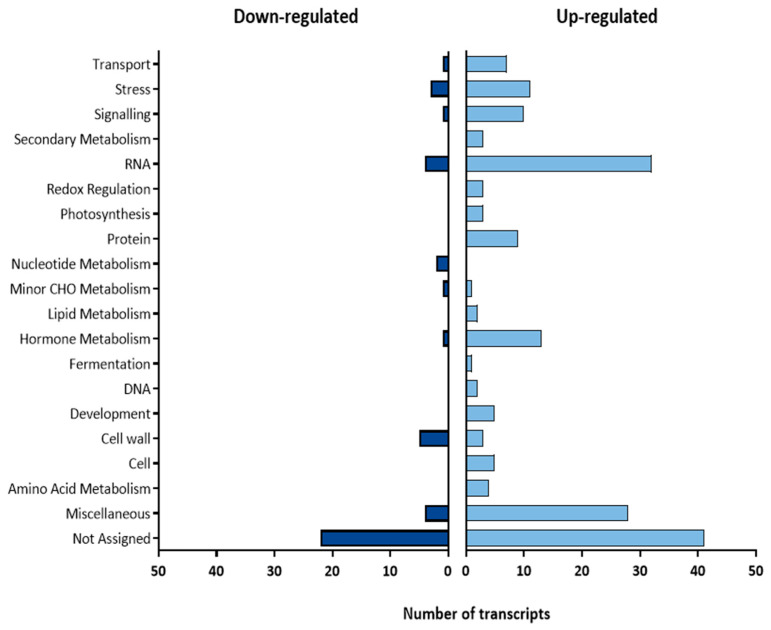
Functional classification of transcripts with differential expression between tomato cultivars, detected in the absence of nematode infection. Up-regulated: transcripts expressed at least double (fold-change ≥ 2) in Motelle than in Moneymaker. Down-regulated: transcripts expressed at least half (fold-change ≤ −2) in Motelle than in Moneymaker. In some cases, a transcript may be included in more than one functional category.

When transcriptomes of uninfected Motelle and Moneymaker plants were compared, 224 differentially expressed transcripts were obtained. Of them, 180 transcripts were significantly more expressed in Motelle than in Moneymaker (up-regulated by at least double), whereas 44 transcripts were less expressed (down-regulated by at least half) in Motelle compared to Moneymaker (Supplemental Data: Table S1). These transcripts were classified by MapMan into different categories according to their biological function (Figure 1).

#### 2.1.1. Transcripts More Expressed in Uninfected Motelle than in Moneymaker

From the 180 transcripts more expressed in Motelle, the not-assigned category was the most represented with 41 transcripts. Among them, the transcript Les.74.1.S1_at (FC = 2.41) represents the *Mi-1.2* gene. In the same group, the high overexpression (FC = 32.77) of LesAffx.66316.1.S1_at can be highlighted, although this transcript corresponded to an undescribed gene.

The RNA category included 32 transcripts. Among them Les.3751.1.S1_at, related to the homeodomain protein h52, was expressed approximately 3 times more in Motelle than in Moneymaker. Moreover, 8 other transcripts belonging to the same category (Les.38.1.S1_at, Les.5091.1.S1_at, Les.3676.1.S1_at, Les.41.1.S1_at, Les.5017.1.S1_at, Les.5017.1.A1_at, Les.4982.1.S1_at and Les.3693.1.S1_at) were related to the MYB transcription factor family.

The miscellaneous category grouped 28 transcripts, including Les.438.1.S1_at (FC = 2.09) associated with the *LEJA1* gene, which codes for jasmonic acid. Moreover, we found a transcript related to the endo-1,4-beta-glucanase precursor (Les.3667.1.S1_at; FC = 2.30), which has a role in cell cycle.

The hormone metabolism category included 13 transcripts; standing out was Les.3679.1.S1_at (FC = 2.39), which corresponds to the transcriptional ethylene factor ERF1.

The stress category grouped 11 transcripts, including Les.75.1.S1_at (FC = 3.92), which represents the *Mi-1.1* gene. Les.1842.1.S1_at encodes for the *NTGP4* gene and can be also mentioned due to its greatest overexpression (FC = 34.69). Two other transcripts were also remarkable in this category: Les.4693.1.S1_at (FC = 4.43), related to the *P4* gene, coding for PR4 and well known for its involvement in defense, especially against nematodes, and Les.2476.1.S1_at (FC = 4.54), related to a wound-induced protein.

In protein category, transcript Les.5230.1.S1_at showed a high expression value (FC = 8), and it was associated with Phospholipase PLDa1.

#### 2.1.2. Transcripts Less Expressed in Uninfected Motelle than in Moneymaker

For the 44 transcripts down-regulated in Motelle compared to Moneymaker, the not-assigned category had the greatest representation with 22 transcripts. Among them, Les.5432.1.S1_at can be mentioned due to its high FC value (−8.17), as well as Les.266.1.S1_a_at (FC = −2.01) for being related to systemine.

The cell wall category included 5 transcripts, 3 of them related to expansins: LesAffx.5130.1.S1_at (FC = −2.57), LesAffx.5130.2.S1_at (FC = −2.33) and Les.3733.1.S1_at (FC = −2.10).

Among the 4 transcripts of the RNA category, Les.3964.1.S1_at (FC = −2.05) corresponded to a WRKY transcription factor.

The only down-regulated transcript included in the hormone metabolism category was Les.4311.1.S1_at, which presented the highest repression level (FC = −15.17) and was associated with GAI, a protein of the DELLA family.

### 2.2. Transcriptomic Profiles of Compatible and Incompatible Interactions of Tomato-M. javanica at 2 Days Post-Infection

**Figure 2:**
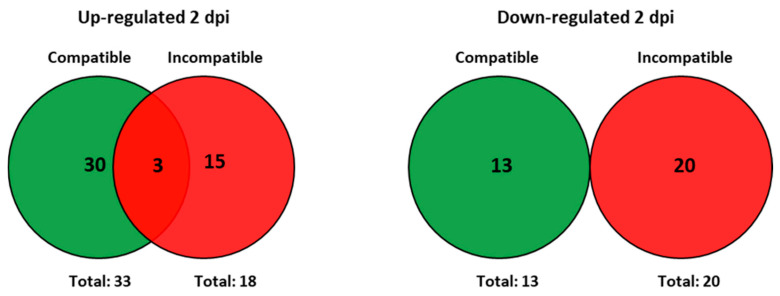
Venn diagrams with the numbers of up- (FC ≥ 2) and down-regulated (FC ≤ −2) transcripts in the microarray analysis depicting differential gene expression (false discovery rate (FDR) < 0.05) in tomato roots, in the compatible (Moneymaker) and incompatible (Motelle) interactions, at 2 days post-inoculation (2 dpi) with *M. javanica*. Data of compatible interactions were obtained by comparing nematode-infected Moneymaker *versus* uninfected Moneymaker. Data of incompatible interactions were obtained by comparing nematode-infected Motelle *versus* uninfected Motelle. Numbers in the overlapping areas represent transcripts common to compatible and incompatible interactions.

At 2 days post-infection (2 dpi) by *M. javanica*, 46 transcripts showed differential expression in the roots of infected plants of Moneymaker compared to uninfected plants of the same cultivar. Of them, 33 transcripts were up-regulated and 13 were down-regulated by nematode infection in this compatible interaction (Figure 2). Regarding the incompatible interaction in Motelle plants, 18 and 20 transcripts were respectively up- and down-regulated by nematode infection at the same time point (Figure 2).

It was notable that the FC values of the up-regulated transcripts reached over 35X in the incompatible interaction, while they did not exceed 5X in the compatible interaction (Supplemental Data: Table S1). On the contrary, no important differences were observed between both interactions regarding the FC values of the down-regulated transcripts, as the maximum was FC = −3.44 in the incompatible and −3.24 in the compatible interaction.

#### 2.2.1. Differential Transcripts Common to Both Compatible and Incompatible Interactions (2 dpi)

At 2 dpi, only 3 up-regulated transcripts were common to both the compatible and incompatible interactions (Figure 2). None of the down-regulated transcripts were common to both interactions at this time point. A description of the biological function and FC values of the 3 common transcripts is shown in Table 1.

Each of these common transcripts presented similar expression values in the compatible and incompatible interactions. Les.5916.1.S1_at encodes the factor ABZ1 (anaerobic basic leucine zipper protein) and was involved in the regulation of transcription. Les.2001.1.S1_s_at was related to the regulation of transcription and LesAffx.69992.1.S1_at was classified in the Amino Acid Metabolism category and associated with a methionine gamma-lyase.

#### 2.2.2. Differential Transcripts Exclusive to the Compatible Interaction (2 dpi)

**Figure 3:**
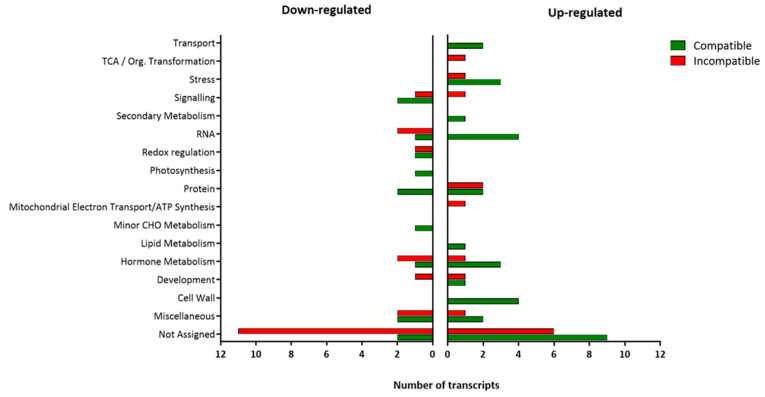
Functional classification of transcripts with differential expression in the infected plants compared to the uninfected plants of the same cultivar, detected exclusively in the compatible (Moneymaker) or in the incompatible (Motelle) interaction at 2 dpi. The transcripts are grouped in biological function categories according to Mapman program. Several transcripts can be included in more than one functional category.

Other differentially regulated transcripts, exclusive to the compatible or the incompatible interaction, were classified according to their biological function as shown in Figure 3. The Not Assigned category included more transcripts than any other for both interactions.

Some of the 30 up-regulated transcripts exclusively detected in the compatible interaction (Figure 2) belonged to cell wall, hormone metabolism, RNA, stress and transport functional categories (Figure 3). The transcript Les.2476.1.S1_at, which showed the highest FC value (4.02), was classified in the Stress category and depicted a gene encoding a wound-induced protein. All 4 transcripts included in the Cell Wall category were associated with the UGE (UDP-glucose 4-epimerase) synthesis, with transcript Les.3267.2.S1_a_at, showing the highest expression value (FC = 3.05).

In the hormone metabolism category, Les.3642.1.S1_at (FC = 2.46) and Les.3551.1.S1_at (FC = 2.24) were associated with ethylene, more specifically with ACC synthase and ER24 (an ethylene-responsive transcriptional coactivator), respectively. In the same category, transcript Les.3668.1.S1_at (FC = 2.35) was associated with a lipoxygenase involved in Jasmonic acid synthesis-degradation process.

In the RNA category, Les.4102.1.S1_at (FC = 2.40) could be mentioned due to its association with ERF2 (ethylene response factor 2). Finally, in the Not Assigned category a transcript (LesAffx.8850.1.S1_at, FC = 2.28) was related to a subtilisin-like protease (sbt4a).

Most of the 13 transcripts down-regulated only in the compatible interaction (Figure 2) were grouped into Not Assigned, Signaling, Miscellaneous, Protein or Redox categories (Figure 3). Among them, Les.3064.1.S1_at is specially mentioned as it showed the higher Fold Change value (FC = −3.24). This transcript, classified in the Not Assigned functional category, was associated with gen *AGP-S1*, which encodes the ADP-glucose pyrophosphorylase large subunit.

The only down-regulated transcript included in the Redox category (LesAffx.66657.1.S1_at) showed the second higher Fold Change value (FC = −3.01). This transcript was related to a hypothetical protein of *Vitis vinifera* and it corresponds to glutaredoxin function.

Les.4383.1.S1_at (FC = −2.03), involved in calcium signaling, could be noted in the Signaling category, as well as Les.3034.1.S1_at (FC = −2.62), a transcript included in the Protein category, which was related to a trypsin proteinase inhibitor precursor.

With regard to the Miscellaneous category, Les.2325.1.S1_at (FC = −2.23) was related to an alcohol dehydrogenase homolog.

#### 2.2.3. Differential Transcripts Exclusive of the Incompatible Interaction (2 dpi)

Among the 15 transcripts up-regulated only during the incompatible interaction (Figure 2), 6 were included in the not assigned category and 2 in the protein category, with the rest of the categories only presenting one transcript each (Figure 3). Regarding the not-assigned category, the highest differential expression was observed in transcript Les.3397.2.A1_at (FC = 35.44), which, together with Les.3397.1.S1_at (FC = 21.64) and Les.3281.1.S1_at (FC = 19.69), was related to a family of trypsin and protease inhibitor proteins.

The two transcripts included in the protein category, Les.4022.1.S1_at (FC = 6.27) and LesAffx.62131.1.S1_at (FC = 2.13), were respectively associated with a proteinase inhibitor and to a L-allo-threonine aldolase-related protein. Moreover, Les.796.1.A1_at (FC = 3.67) and Les.2832.1.S1_at (FC = 2.45) participated in carbonic anhydrase and peroxidase production, respectively.

Transcript Les.122.1.S1_at (FC = 2.65) was included in stress biotic category and encoded for a chitinase.

Regarding the 20 transcripts representing genes down-regulated exclusively in the incompatible interaction (Figure 2), the hormone metabolism, RNA and miscellaneous categories presented 2 transcripts each. The redox regulation, signaling and development categories included only one transcript each, while the not-assigned category grouped 11 transcripts.

In the RNA category, the transcript LesAffx.9910.1.S1_at (FC = −3.44) was related to WRKY transcription factors.

Les.3464.1.S1_at (FC = −3.26) was included in the hormone metabolism category and associated with the *EXT1* gene, which participates in the synthesis of an extensin-like protein Ext1. In this category, the lipoxygenase precursor Les.3632.1.S1_at (FC = −2.38) is also included, involved in the jasmonic acid synthesis-degradation process.

Among the transcripts grouped in the not-assigned category, Les.5956.2.S1_at (FC = −2.00) was related to a proline deshydrogenase inducible by cold stress, and Les.4335.1.S1_at (FC = −2.02) corresponded to PR-P2 (pathogenesis-related protein P2).

### 2.3. Transcriptomic Profiles of Compatible and Incompatible Interactions of Tomato-M. javanica at 12 Days Post-Infection

**Figure 4:**
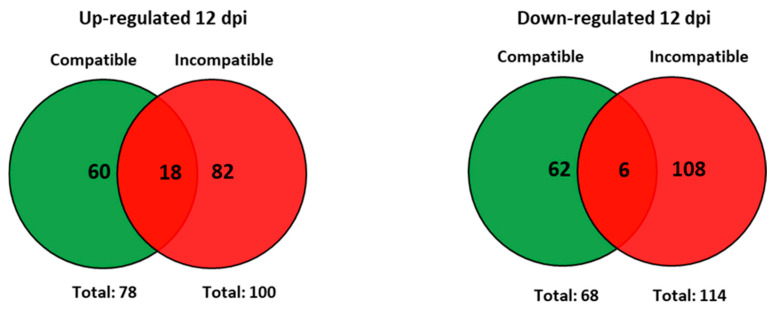
Venn diagrams with the numbers of up- (FC ≥ 2) and down-regulated (FC ≤ −2) transcripts in the microarray analysis depicting differential gene expression (false discovery rate (FDR) < 0.05) in tomato roots, in the compatible (Moneymaker) and incompatible (Motelle) interactions, at 12 days post-inoculation (12 dpi) with *M. javanica*. Data of compatible interactions were obtained by comparing nematode-infected Moneymaker with uninfected Moneymaker. Data of incompatible interactions were obtained by comparing nematode-infected Motelle with uninfected Motelle. Numbers in the overlapping areas represent transcripts common to compatible and incompatible interactions.

At 12 days post-infection (12 dpi) by *M. javanica*, 146 transcripts showed differential expression in the roots of infected plants of Moneymaker compared to uninfected plants of the same cultivar. Of them, 78 transcripts up-regulated and 68 down-regulated were obtained at this time point of the compatible interaction (Figure 4). For the incompatible interaction (infected vs. uninfected roots of Motelle), 100 and 114 transcripts were respectively up- and down-regulated at the same time point (Figure 4).

The FC values of differential transcripts in the incompatible interaction (15.41 to −47.32) were by far higher than those in the compatible interaction (4.83 to −4.18).

#### 2.3.1. Differential Transcripts Common to Both Compatible and Incompatible Interactions (12 dpi)

At 12 dpi, 18 up-regulated and 6 down-regulated transcripts were common to both compatible and incompatible interactions (Figure 4). Differential expression (FC values) of the 18 common up-regulated transcripts was similar for both compatible and incompatible interactions, while most of the 6 common down-regulated transcripts were more repressed in the incompatible than in the compatible interaction (Table 2).

The 18 common up-regulated transcripts were classified in 7 functional categories: not assigned, RNA, miscellaneous, hormone metabolism, lipid metabolism, cell cycle and development.

From the 5 transcripts grouped in the not-assigned category, Les.4596.1.S1_at represents a defensin protein involved in plant protection, the 60S ribosomal protein (ACI29) in particular (Supplemental Data: Table S1), with its expression being higher in the incompatible interaction (FC = 5.27) than in the compatible one (FC = 3.46).

Four transcripts of the RNA category were detected. Of them, Les.41.1.S1_at and Les.5442.1.S1_at were associated with a MYB transcription factor and to an Aux/IAA transcription factor, respectively. In the same category, Les.83.1.S1_at was related to the *Kn-1* gene (Homeotic protein knotted-1), a transcription factor that regulates genes involved in development.

Among the 2 transcripts in the Hormone metabolism category, Les.2504.1.A1_at, involved in salicylic acid regulation, can be highlighted.

The 3 transcripts in the Miscellaneous category included LesAffx.30937.1.S1_at and LesAffx.9038.1.S1_at, both related to the cytochrome P450 monooxygenase.

The only transcript included in the Lipid Metabolism category, Les.4333.1.S1_at, was related to lipid transport and specifically with the TSW12 protein.

The 6 down-regulated transcripts common to both interactions at 12 dpi were classified into 4 categories: Miscellaneous, Transport, Stress and Signaling. The Miscellaneous category included Les.228.1.S1_a_at and Les.228.1.S1_at, both associated with a putative proline-rich protein. The third transcript in the same category, LesAffx.21576.1.S1_at, was related to an extensin-like protein. All these 3 transcripts were more repressed in the incompatible than in the compatible interaction (Table 2).

The only transcript included in the Stress category, LesAffx.64062.1.S1_at, was related to a germin-like protein. It was more strongly repressed in the incompatible than in the compatible interaction, just like what happened to Les.5112.1.S1_at, which was included in the Transport category and associated with a silicon transporter.

The only down-regulated common transcript in the Signaling category was associated with a protein containing an EF-Hand motif. The differences in expression between infected and uninfected plants at 12 dpi were very similar in both compatible and incompatible interactions (Table 2).

#### 2.3.2. Differential Transcripts Exclusive to the Compatible Interaction (12 dpi)

In addition to the common transcripts, when infected plants were compared to uninfected plants at 12 dpi, other transcripts differentially regulated were detected exclusively in the compatible or in the incompatible interaction (Figure 4).

**Figure 5:**
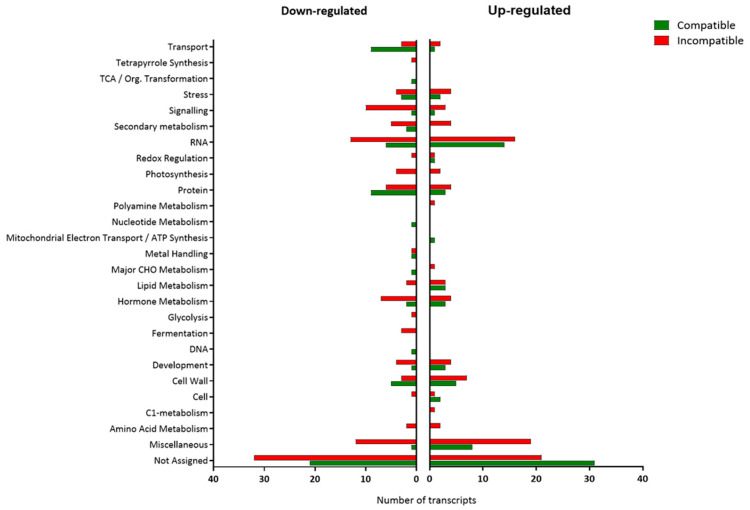
Functional classification of transcripts with different expression in the infected plants than in the uninfected plants of the same cultivar, detected exclusively in the compatible or in the incompatible interaction at 12 dpi. The transcripts are grouped into biological function categories according to Mapman program. Several transcripts can be included in more than one functional category.

A total of 60 up- and 62 down-regulated transcripts were detected exclusively in the compatible interaction and classified according to their biological function (Figure 5).

The 60 up-regulated transcripts, exclusive to the compatible interaction, were grouped into 14 functional categories (Figure 5): Not assigned, RNA, Cell Wall, Miscellaneous, Protein, Development, Hormone Metabolism, Lipid metabolism, Stress, Cell, Redox, Signaling, Transport and Mitochondrial electron transport.

Les.417.1.S1_at presented the greatest FC value (4.83) among the transcripts up-regulated at this time point exclusively in the compatible interaction. It was included in the Hormone Metabolism category and related to the *snakin-1* gene, which is involved in redox balance, growth, development and plant resistance.

Several other transcripts up-regulated at 12 dpi in the compatible but not in the incompatible interaction were assigned to the Cell Wall category, with the function of degradation or modification.

Of the transcripts included in the Not Assigned category, Les.3559.1.A1_at (FC = 3.22) was related to a Gamma-thionin.

In the Stress category, two transcripts were related to plant defense: Les.2476.1.S1_at (FC = 3.17), associated with a wound induced protein, and Les.3583.1.A1_at (FC = 2.93), related to the TSI-1 protein.

Les.14.1.S1_at (FC = 2.78), in the Protein category, and LesAffx.8850.1.S1_at (FC = 2.19), in the Not Assigned group, were respectively associated with the *sbt2* and *sbt4a* genes, coding for two proteases.

In the RNA category, several transcripts were associated with the regulation of transcription. Among them, Les.3706.1.S1_at (FC = 2.90) and Les.4087.1.S1_at (FC = 2.70) were associated with proteins classified in the Aux/IAA family.

The Miscellaneous category included transcript Les.438.1.S1_at (FC = 2.25), with participation in the synthesis of jasmonic acid (LEJA1) and, therefore, implicated in defense against pathogens.

Finally, transcript Les.38.1.S1_at (FC = 2.56), in the RNA category, was associated with a myb-related transcription factor (THM16).

The 62 transcripts down-regulated at 12 dpi exclusively in the compatible interaction were classified into different functional categories, standing out the Not Assigned, Transport, Protein and RNA categories, among others.

In the Not Assigned category, Les.100.1.S1_at (FC = −4.13), was associated with the Dif10 extensin-like protein), and Les.3601.1.S1_s_at (FC = −2.93), was related to an extensin class I protein.

Among the 9 transcripts included in the Transport category, Les.102.1.S1_at (FC = −4.18) was related to the *LePT2* gene, an inorganic phosphate transporter, and LesAffx.286.9.S1_at (FC = −3.13) was associated with a protein similar to aquaporin.

Of the 3 transcripts grouped in the Stress category, Les.3582.1.S1_at (FC = −3.13) was related to the *Mdip1* gene implicated in the germin-like protein synthesis (GLP).

Les.2307.1.A1_at (FC = −3.06), the unique transcript included in the category of TCA/Org. Transformation, was associated with the succinate dehydrogenase (SDH).

In the Hormone metabolism category, Les.3466.1.S1_at (FC = −2.21) and LesAffx.8333.1.S1_at (FC = −2.11) were related to jasmonate and ethylene regulation, respectively.

#### 2.3.3. Differential Transcripts Exclusive to the Incompatible Interaction (12 dpi.)

A total of 82 up-regulated and 108 down-regulated transcripts were detected at 12 dpi. in the incompatible but not in the compatible interaction (Figure 4).

Regarding the 82 up-regulated transcripts, these were classified into 19 categories (Figure 5), among which the following are noteworthy: Not Assigned, Miscellaneous, RNA, Cell Wall, Development, Hormone metabolism, Protein, Secondary metabolism and Stress.

Les.4685.1.S1_at, included in the Not Assigned category with the greatest number of transcripts, presented the highest value expression (FC = 15.41) and was related to a hyoscyamine 6-dioxygenase. Other transcripts of this category were Les.3974.1.A1_at (FC = 4.90), related to a metallocarboxypeptidase inhibitor, and Les.804.1.S1_at (3.94), remarkable for its essential defense function in synthesizing the hypersensitive response-assisting protein (HRAP).

A group of transcripts were associated with different defense molecules against pathogens: Les.3668.1.S1_at (FC = 4.78) from the Hormone Metabolism category was related to lipoxygenase A; Les.4911.1.S1_at (FC = 8.05) from the Secondary Metabolism category was related to chalcone synthase B; Les.3408.1.S1_at (FC = 6.42) from the Stress category was associated with pathogenesis-related protein leaf 6 (P6), and the transcript Les.3683.1.S1_at (FC = 3.97) was associated with pathogenesis-related protein 5 (PR-5x).

Transcript Les.3525.1.S1_at (FC = 4.88) from the Amino Acid metabolism category was associated with ornithine decarboxylase, and Les.172.2.S1_a_at (FC = 2.38), in the Polyamine metabolism category, was related to the SPM1 protein.

Also in the Stress category, LesAffx.48086.1.S1_at (FC = 3.73) was up-regulated and related to a gene encoding for the antimicrobial peptide snakin-2 (SN2).

Concerning the RNA category, the most notable was Les.3814.1.S1_at (FC = 2.97), related to the *fer* gene, which codifies a bHLH transcription factor.

LesAffx.69609.1.S1_at (FC = 2.57) intervened in salicylic acid metabolism through the production of S-Adenosyl-L-Methionine: Salicylic Acid Carboxyl Methyltransferase enzyme (SAMT).

In the photosynthesis-specific (PS) category, the overexpression of Les.376.1.S1_at (FC = 3.35) was remarkable as this transcript was associated with the Rubisco small subunit precursor (RbCS).

The Protein category was represented by Les.513.1.S1_at (FC = 4.21), related to a gene encoding the subtilisin-like protease. Moreover, Les.3972.1.S1_at (FC = 2.30) in the Cell Wall category was associated with expansin 9.

Regarding the 108 transcripts down-regulated at 12 dpi, but exclusively in the incompatible interaction, the functional category that included the greatest number of transcripts was Not Assigned, with 32 transcripts (Figure 5).

Les.5917.1.S1_at, included in the Hormone Metabolism category, could be emphasized due to its high FC value (−47.32) and for its association with 1-aminocyclopropane-1-carboxylate oxidase (ACO5) and ethylene biosynthesis regulation. Another transcript involved in the ethylene pathway was Les.3818.1.S1_at (FC = −3.22), which coded to an EREB. Also in the Hormone Metabolism category, Lipoxygenase was represented by transcript Les.3632.1.S1_at (FC = −2.07).

In the RNA category, 3 transcripts were found related to the APETALA2/Ethylene-responsive element binding factor (AP2/ERF) protein family. Specifically, Les.4102.1.S1_at (FC = −3.39), encoding for ethylene response factor 2 (ERF2); LesAffx.20450.1.S1_at (FC = −2.27), involved in transcriptional regulation; and LesAffx.56.2.A1_at (FC = −2.03), related to a senescence-associated protein. Another two down-regulated transcripts in RNA category were Les.3707.1.S1_at (FC = −2.95) and LesAffx.71035.1.S1_at (FC = −2.68), related to IAA2 protein and to putative auxin-induced SAUR-like protein, respectively, both auxin-response factors (ARFs).

In the Miscellaneous category, transcripts LesAffx.66472.1.S1_at (FC = −8.15) were associated with the defense response, as they were related to lipid transfer proteins (LPTs).

In the Miscellaneous category, transcript LesAffx.66472.1.S1_at (FC = −8.15) was associated with the defense response, as it was related to a lipid transfer protein (LPT).

Les.27.1.S1_at (FC = −6.34) was grouped in the Secondary Metabolism category and related to the copalyl diphosphate synthase (CPS) of terpenoids metabolism.

In the Stress category, the transcript Les.2137.1.S1_at (FC = −3.72) codes for receptor-like protein EIX1.

The Signaling category included LesAffx.16424.1.S1_s_at (FC = −2.25), LesAffx.16424.1.A1_at (FC = −2.24) and Les.4316.1.S1_at (FC = −2.01), all related to a gene encoding mitogen-activated protein kinase 3 (MPK3).

### 2.4. Comparison Between the Earlier (2 dpi) and the Later (12 dpi) Stages of Infection by M. javanica

**Figure 6:**
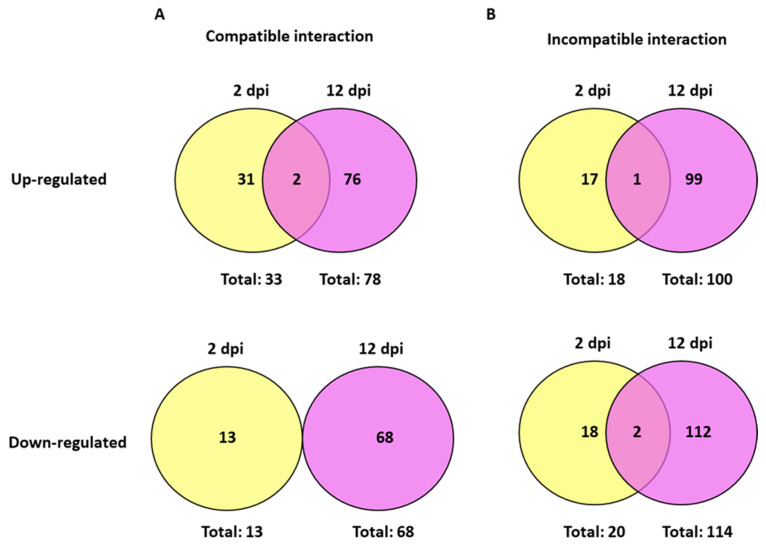
Venn diagrams with the numbers of up- (FC ≥ 2) and down-regulated (FC ≤ −2) transcripts in the microarray analysis depicting differential gene expression (*p* < 0.05) in tomato roots in the compatible (**A**) and incompatible interactions (**B**), at 2 or 12 days post-inoculation with *M. javanica*. Data of compatible interactions were obtained by comparing nematode-infected Moneymaker with uninfected Moneymaker. Data of incompatible interactions were obtained by comparing nematode-infected Motelle with uninfected Motelle. Numbers in the overlapping areas represent transcripts common to both time points. For each treatment, RNA from three biological replicates was used, each one obtained by pooling the whole root system of three plants.

The total number of transcripts showing a differential expression between infected and uninfected plants was higher at 12 dpi than at 2 dpi for both compatible (146 vs. 46) and incompatible (214 vs. 38) interactions. The same trend was maintained for the up-regulated transcripts in compatible (78 vs. 33) and incompatible (100 vs. 18) interactions, as well as for the down-regulated transcripts in compatible (68 vs. 13) and incompatible (114 vs. 20) interactions (Figure 6).

For each of the compatible or incompatible interactions, most of these transcripts were differentially expressed only at 2 dpi or at 12 dpi, and they can be considered exclusive to one or another stage of infection. Consequently, information about them was previously detailed in the specific section for each time point of the corresponding compatible or incompatible interactions.

As also shown in Figure 6, only two transcripts were up-regulated at both 2 and 12 dpi during the compatible interaction. Regarding the incompatible interaction, one up-regulated and two down-regulated transcripts were common to both time points (Figure 6). These common transcripts are described in Table 3.

During the compatible interaction (Moneymaker plants), two transcripts were up-regulated by the nematode infection both at 2 and 12 dpi: Les.2476.1.S1_at, which was related to a wound-induced protein and was involved in the abiotic stress response, and LesAffx.8850.1.S1_at, related to a subtilisin-like protease (sbt4a). The relative expression values of these transcripts were slightly higher in the earlier than in the later stage of infection (Table 3). None of the transcripts down-regulated during the compatible interaction were common to both time points (Figure 6).

In the incompatible interaction (Motelle plants), one up-regulated transcript was common to both time points (Table 3): LesAffx.69992.1.S1_at was described as a predicted protein and involved in methionine synthesis. This transcript presented a higher FC value at 12 dpi (FC = 3.27) than did at 2 dpi (FC = 2.16).

Two down-regulated transcripts showed very similar FC values for both 2 and 12 dpi of the incompatible interaction (Table 3): LesAffx.3253.2.S1_at was related to ABA 8’ hydroxylase, while Les.3632.1.S1_at corresponded to the hormone metabolism category and specifically to a lipoxygenase (*loxD*).

### 2.5. Validation of Microarray Data 

The relative expression values of the transcripts analyzed by qRT-PCR are shown in Table 4.

**Figure 7:**
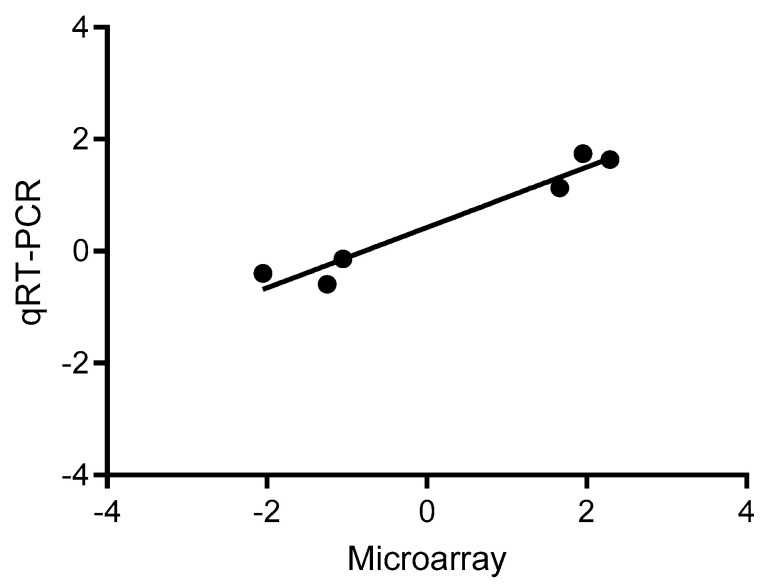
Correlation between relative expression (Log_2_ RE) data obtained from microarray analysis (X axis) and qRT-PCR (Y axis), with a statistically significant (*p* = 0.0011) Pearson correlation coefficient value (r = 0.9729).

A positive correlation between qRT-PCR data and those previously obtained by microarray analysis was obtained (Figure 7), with a Pearson correlation coefficient (r) value of 0.9729, which was statistically significant (*p* = 0.0011), therefore validating the results obtained by microarray analysis.

## 3. Discussion

In this study, we employed Affymetrix microarrays to profile gene expression in tomato, a species for which large scale transcriptomic resources generated by both microarray and RNA seq platforms already exist. Although RNA seq has become the dominant technology in recent years, extensive cross-platform comparisons show that microarrays produce highly comparable expression profiles, with correlation coefficients above 0.8 and substantial overlap in differentially expressed genes detected by both platforms, confirming the validity of microarrays for genome wide expression studies [54,55]. Microarray-based transcriptome datasets, such as the comprehensive Affymetrix profiling of gene expression across 10 stages of tomato fruit development [56], demonstrated that microarrays reliably capture biologically meaningful transcriptional programs and remain widely used in functional genomics of this crop. Similarly, large-scale RNA seq profiling under exogenous ABA treatment reinforce the consistency of global expression patterns previously captured using microarrays [57]. Furthermore, integrated tomato omics resources, such as TOMATOMICS, combine Micro Tom full length cDNAs, Heinz 1706 RNA seq, and microarray datasets, illustrating that microarray data remain interoperable and biologically consistent within the broader tomato transcriptomics framework [58]. Collectively, these findings support that our Affymetrix-based analysis yields biologically robust and reliable expression measurements in tomato, fully comparable to what would be obtained with RNA seq, particularly for differential expression analyses and well expressed transcripts.

This is the first time that microarrays have been used to analyze the transcriptome of the whole root tissue of adult tomato plants infected by root-knot nematodes. In previous works with these and other nematodes, microarray analysis was performed in younger plants: 5-week-old plants [53], plants 3–4-weeks post-germination [51], or even newly germinated tomato seedlings [43,47]. Although resistance to nematodes mediated by the *Mi-1* gene in tomato is active soon after plant germination [59], whereas it is developmentally regulated against aphids [60] and whiteflies [61], it is important to note this methodological novelty of the present work. Moreover, some of the previous studies did not consider the whole root tissue as they focused on just 1.5 cm or less of root length to include some particular fragments closely related to the formation of giant cells after nematode infection [43,47].

Unlike other works where only compatible tomato–nematode interaction was analyzed [43,46,47], the possibilities of microarray analysis have been exploited further in the present study, expanding the focus also to the incompatible interaction and comparing both interactions, similarly to [41] when they analyzed the resistance to cyst nematode *Globodera rostochiensis* mediated by the *Hero A* gene. In another work [51], the same tomato cultivars were compared as in the present study, but their young plants were not infected with *M. javanica*, but with *M. hapla* and *M. incognita*. Also, Ref. [53] compared the compatible and incompatible interactions of tomato with root-knot nematodes, but they worked with *M. incognita* and analyzed younger plants, as mentioned above.

To our knowledge, only one similar comparison using microarray technology had been made between Motelle and Moneymaker tomato roots in the absence of any infestation, but that study only analyzed the expression of 1547 genes which did not include the *Mi-1* gene [51], unlike the present study which analyzed the expression of approximately 9200 genes. In most previous works, microarrays were designed to identify specific plant–pathogen interactions [46] or a particular physiological event [1]. Conversely, a commercial matrix was used in the present analysis where cDNA clones were not specifically designed for the study of the nematode infection, so this can be considered an unbiased study, similar to the work of [41]. The same methodological approach used in the present study was employed by our group in previous works to compare, at the transcriptomic level, the leaf tissues of uninfested plants of Motelle and Moneymaker cultivars [38], as well as the resistant and susceptible responses of tomato to the attack by the whitefly *B. tabaci* [62].

### 3.1. Basal Differences in the Transcriptional Profiles of Uninfected Roots of Motelle and Moneymaker

When compared to Moneymaker, the presence of *Mi-1* in uninfected roots of Motelle is accompanied by the differential expression of other genes related to the defense response. Motelle and Moneymaker are nearly isogenic tomato cultivars differing only in a 650 kb fragment of chromosome 6, including the *Mi-1* gene in Motelle [63]. As it was expected, transcripts encoding for *Mi-1.1* and *Mi-1.2* were more expressed in Motelle than in Moneymaker, which confirms the constitutive expression of the resistance gene *Mi-1* in roots of the Motelle cultivar [18,64]. However, the expression of *Mi-1* was not the only basal difference observed, as more than 200 transcripts with differential expression were detected. This finding was in contrast with the results obtained by [51], where only one gene (encoding a glycosyltransferase associated with plant stress and defense response) exhibited a significant cultivar-dependent difference in gene expression in the absence of infection. However, methodological differences between the two studies must be taken into account: the type of microarrays used, age of the plants analyzed, etc.

In the present analysis, the number of transcripts more expressed in uninfected Motelle than in Moneymaker was at least 4 times greater than those down-regulated. Moreover, the differences in their relative expression levels were also notable, with the highest FC value observed in the up-regulated transcripts, twice that of the biggest value recorded in the down-regulated transcripts. This suggests that, even in the absence of infection, the genomic environment is a priori more active in Motelle than in Moneymaker, allowing defense genes in the resistant cultivar to recognize potential pathogens faster and in greater magnitude than in the susceptible cultivar, and probably to develop a more effective defense at the interaction time [39,65]. In addition, the functions of many of the transcripts more expressed in Motelle than in Moneymaker were related with RNA transcription and regulation, hormone metabolism, stress and protein, all functions related to plant defense.

In the RNA functional category, a transcript encoding for the homeodomain protein h52 was approximately 3 times more expressed in Motelle than in Moneymaker. This protein is usually up-regulated upon infection with virulent pathogens; it is a transcription factor involved in plant defense as it is essential for cell protection by limiting the programmed cell death, and is necessary to regulate the extension of the hypersensitive response [66]. Leaving aside the RNA group, most transcripts were included in the not-assigned and miscellaneous categories, which often have the greatest representation in studies that follow a methodology similar to that used in this case [38,47].

The transcript with the highest expression value in the Stress category was related to the gene encoding the geranylgeranylated protein 4 (NTGP4) of *Nicotiana tabacum*. The same transcript also showed very high differential expression when comparing uninfested leaf tissues of Motelle and Moneymaker tomato [38]. The *NTGP4* gene participates in processes of response to biotic stimuli as it was induced by nematode infection in the roots of Moneymaker [47]. Furthermore, *NTGP4* probably contributes to the salt tolerance of another tomato cultivar, as higher basal expression of the gene was observed in the leaves of the tolerant tomato compared to the sensitive Moneymaker, although *NTGP4* was not responsive to salt stress in either tomato genotype [67].

Another transcript included in the Stress functional category was more than 4 times more expressed in Motelle than in Moneymaker and it was associated with the *P4* gene, which encodes for the PR-4 protein (pathogenesis-related protein 4). PR-4 is known for inducing defense in several plant–pathogen interactions and especially for its antifungal role in wheat [68]. However, its implication during the plant–*Meloidogyne* interaction is not clear: In Arabidopsis, Ref. [45] detected the induction of PR-4 by *M. incognita* but, at the contrary, other authors did not detect changes in the expression of PR-4 in Arabidopsis [69] or in soybean although other PRs were differentially expressed following infection by *M. incognita* [48]. Another study had shown that PR-4 was induced by exogenous application of jasmonic acid (JA) and ethylene (ET) in wheat [68]. Moreover, this *P4* gene is involved in SA pathway activation in tomato infestation with aphids [70] and bacterial infection [71]. Moreover, whitefly feeding on tomato leaves induces PRs, including the *P4* gene, among others [72]. In the absence of infection, resistant plants would have activated the SA route, which could be essential for the resistance against nematodes [73]. This agreed with our results where LEJA-1 (Jasmonic Acid 1) and ERF-1 (an ethylene response factor) were also up-regulated in uninfected Motelle compared to Moneymaker. The overexpression in Motelle roots of *ERF1* and *LEJA1* genes, which are both necessary to regulate basal and R-gene-mediated defense responses against pathogens and insects [74,75], confirms that both hormones, ET and JA, seem to work synergistically [47,76]. However, Bhattarai et al. [45] concluded that JA-dependent signaling does not play a role in *Mi-1*-mediated defense to RKNs, although an intact JA signaling pathway is required for tomato susceptibility. Moreover, *Mi-1*-mediated resistance to *M. incognita* in tomato may not depend on ethylene [65]. On the other hand, JA and ET signaling pathways contribute to plant defense against necrotrophic pathogens [77]. The results presented so far indicate together that uninfected Motelle plants would be more protected a priori than Moneymaker plants against different types of pathogens (biotrophics and necrotrophics).

In the present study, a gene encoding an endo-1,4-beta-glucanase precursor (*Cel1*) was expressed twice as much in uninfected roots of Motelle as in those of Moneymaker. However, it was previously demonstrated that endo-β-1,4-glucanases Cel1 and Cel2 are involved in the susceptibility to *Botrytis cinerea* in tomato [78]. Endo-β-1,4-glucanases are required for the degradation of the cell wall primary septum by contributing to the crystallization process in Arabidopsis [79]. Cell wall modifications in the feeding cells during the compatible tobacco–nematode interactions are due to cell wall-modifying enzymes of plant origin, and not nematode origin [80].

Another transcript in the Protein category and related to a gene encoding Phospholipase PLDa1 was 8-fold more expressed in Motelle than in Moneymaker in the absence of infestation. This protein is the most abundant PLD in Arabidopsis [81], involved in biotic and abiotic stress responses, including plant–microbe interactions, wounding, freezing, dehydration and salinity [82,83,84]. In tomato, PLDα1 has a ubiquitous presence in the roots, stems, petioles, leaves, flowers and fruit of mature plants [85], but the basal expression level is low in roots and leaves [86]. PLDα1 of tomato is an ortholog of PLDα1 of Arabidopsis, and exhibits very strong responses to salt and drought stress [86].

Regarding the function of the transcripts less expressed in uninfected Motelle than in Moneymaker, RNA and Cell Wall were the most represented categories, although with a lower number of genes than those up-regulated from the same categories. In the Cell Wall category, the reduced expression in Motelle of a transcript associated with an expansin can be highlighted, along with two others associated with a beta expansin precursor. It was demonstrated that a low expression of a plant-origin expansin reduces the ability of *M. javanica* to complete its life cycle in tomato roots [87]. So, the low basal levels of expansin detected in Motelle would contribute to impair the successful nematode–plant interaction that occurs in the susceptible Moneymaker. Repression of particular gene subsets, such as those involved in defense and secondary metabolism, usually occurs in pathogenic and symbiotic interactions [88,89,90,91].

The highest negative ER value among all transcripts down-regulated in Motelle was represented by the *GAI* gene, which encodes the Gibberellic acid-insensitive mutant protein (GAI), included in the DELLA group of proteins [92]. DELLAs restrict plant growth by suppressing the action of GAs [93]. Reciprocally, GAs regulate growth through the degradation of DELLA proteins [94,95,96]. Motelle had also previously shown in the leaf tissues much lower basal expression levels of the same gene than those of Moneymaker [38]. This might suggest that at least this particular GAI protein can play a role in the *Mi-1*-mediated resistance of tomato against both nematodes and whiteflies. In Arabidopsis and tomato, DELLA proteins control plant defense to pathogens by modulating the responses dependent on SA and JA [97,98,99]. In any case, the role of the GA signaling pathway in plant defense is ambiguous as antagonistic effects have been obtained from different studies [100].

Another transcript less expressed in Motelle than in Moneymaker was related to WRKY transcription factor 10. It is noteworthy that other transcription factors showed the opposite situation, as they appeared to be more expressed in Motelle than in Moneymaker. Various WRKY transcription factors play a central role in transcriptional reprogramming associated with plant immune responses [101]. However, the contribution of the products of individual *WRKY* genes to plant interactions with specific pests or pathogens is subtle, probably functioning as elements of a transcriptional network composed of feedback loops and feed-forward modules [102].

Another down-regulated transcript represented a gene encoding systemin, an essential regulatory component of wound-induced systemic defense responses in tomato [103]. A mechanism was recently discovered by which two antagonistic systemin receptors (SYR1 and SYR2) of tomato act in concert to initiate and attenuate systemic wound responses, which is important since sustained activation of wound signaling can inhibit plant growth and fitness [104].

These results show that, despite being nearly isogenic cultivars, there are many basal differences between the roots of Motelle and those of Moneymaker even before nematode infection. The genes represented by these differential transcripts could be considered in principle as good candidates to participate in the resistance to nematodes mediated by the *Mi-1* gene, although to clarify their relevance, it is necessary to analyze their evolution after the nematode attack during both compatible and incompatible interactions.

### 3.2. Tomato Transcriptome Reprograming Due to Infection by M. javanica

The comparison of plants infected by *M. javanica* with non-infected plants of the same cultivar confirms that nematode infection induces transcriptional reprograming in tomato roots during both compatible and incompatible interactions, in agreement with previous results by other authors [43,51].

In the earlier phase analyzed in this study (2 dpi), the number of transcripts altered by the nematode infection was slightly higher in the compatible than in the incompatible interaction, as it was observed previously [51]. The high amount of transcripts detected at an early phase in the compatible interaction probably corresponded to the establishment of the feeding site [45,105,106]. These results differ from those in other work [47], where more differential genes were obtained in the incompatible than in the compatible interaction at 24 h post-inoculation. However, this and the present study agreed that most differential transcripts were up-regulated and presented higher FC values in Moneymaker than in Motelle.

At the late phase of infection (12 dpi), more differentially regulated genes were observed in the incompatible than in the compatible interaction, in accordance with other results indicating that genes involved in a resistant response are either up- or down-regulated over time [51]. Although that study and the present one considered somewhat different time points for root collection, both agree that activation of the resistant response persists or even increases over the first days after nematode infection. This is also consistent with other previous direct observations [59].

#### 3.2.1. Transcripts Common to the Compatible and Incompatible Interactions with *M. javanica*

Only 3 differential transcripts were common to both compatible and incompatible interactions at 2 dpi and similarly up-regulated after nematode infection. They might be related to the nematode attempts to initiate the establishment of the feeding site at the early stage of infection in the roots of both Motelle and Moneymaker, as previous works indicated [47]. In fact, this theory is reinforced by the functions of these three common genes, as two of them are associated with transcription factors and the third encodes a methionine gamma-lyase, which plays a key role in the regulation of sulphur-containing aminoacids.

Regarding the late phase of infection, two of the 18 up-regulated transcripts common to both compatible and incompatible interactions are associated with a defensin protein, a 60S ribosomal protein (ACI29) and with the cyclin CycD3;3. The overexpression of ribosomal proteins at 7 dpi in giant cells in both Arabidopsis [45] and tomato [43] has been linked to increased protein synthesis activity in the formation of the feeding site. Overexpression of cyclins at 12 dpi in giant cells associated with cell cycle activation has also been described [48,107]. Moreover, a tight control of regulation of CycD3 is required for normal vascular development as part of the mechanism controlling organ growth in higher plants [108]. Therefore, the results obtained in the present study for the compatible interaction would be consistent with the giant cell formation process. In the incompatible interaction, however, this is the first time that overexpression of this type of proteins has been detected, so it would be necessary to further investigate its potential function in relation to resistance.

It is interesting that all 6 transcripts down-regulated at 12 dpi, common to both interactions, showed higher differential expression in Motelle than in Moneymaker, which would indicate greater activity at the transcriptomic level in the incompatible interaction, as previously observed [45]. Two of these common transcripts were associated with a putative proline-rich protein, which plays an important role in plant growth and in cell wall matrix formation [109]. Another common down-regulated transcript was related to an extensin-like protein, extensins being constituents of the cell wall and widely associated with defenses in the root against pathogens [110,111].

#### 3.2.2. Transcriptome Reprograming Exclusive of the Compatible Interaction

##### Early Phase of the Compatible Interaction (2 dpi)

In the early phase of the compatible interaction, one of the main overexpressed functions was that of the stress, and the transcript with the highest differential expression corresponds to a protein related to wounding. This may be due to the mechanical damage caused by the nematode when it penetrates the root, as observed in [112], and that the secretions of nematodes are mainly directed at modifying the structure of the plant cell wall [51].

Four transcripts included in the Cell Wall category were also up-regulated at 2 dpi only in the compatible interaction, being associated with genes related to UGE (UDP-glucose 4-epimerase) synthesis. UGEs are responsible for UDP-galactose synthesis from UDP-glucose in plant cells, and mutation of a UDP-glucose-4-epimerase in Arabidopsis roots causes hypersusceptibility to the cyst nematode *Heterodera schachtii* [113].

Several other genes up-regulated in the early phase of the compatible interaction were associated with ethylene, such as ethylene-responsive transcriptional coactivator 24, or ethylene response factor 2 (ERF2). Another ethylene response factor ERF1) had been more expressed in Motelle roots than in those of Moneymaker, both in the absence of nematode infection. The *ERF1* gene is necessary to regulate basal and R-gene-mediated defense responses against pathogens and insects [74]. ERF1 integrates signals from JA and ET and overexpression of ERF1 results in the activation of many defense-related genes, enhancing resistance to necrotrophic pathogens [74]. However, the role of ERFs varies depending on the pathogen, as overexpression of ERF1 in Arabidopsis causes enhanced susceptibility to *P. syringae* while increasing resistance to *B. cinerea* [114].

The manipulation exerted by the nematode in the earliest phase of the compatible interaction was also evident in the down-regulation of signaling, which was one of the prominent functions repressed during compatible interaction, as pointed out in previous studies [44,65]. In Arabidopsis, the repression of signaling through ion channels and transporters appears to be very pronounced during the establishment of the feeding site by the nematode [44]. On the other hand, and as discussed above, the suppression of some of the hormonal signaling pathways, such as the SA pathway, is part of this process [65]. The identification and description of genes with signaling function may be of interest and provide information on key processes of the tomato–nematode pathogenesis. The gene down-regulated only in Moneymaker but not in Motelle, with the highest level of differential expression, was the one encoding for the major subunit of an enzyme (ADP-glucose pyrophosphorylase) related to sugar levels, particularly starch, and abiotic stress in tomato plants, as it is induced when the plant is exposed to high salinity levels [115]. As in the present analysis, this gene appears to be repressed in the first days after the plant perceives the nematode, which would indicate that it is also related to biotic stress, in addition to abiotic stress.

Certain genes down-regulated in Moneymaker at 2 dpi, but not in Motelle, were related to protein degradation. It should be noted that other genes belonging to the Protein functional category were found up-regulated in both the compatible and incompatible interactions. In previous studies, protein formation also appeared to be overexpressed in both interactions [46,47,116,117]. In the last of these works [47], induction in both interactions justifies the activation of the octadecanoic acid signaling pathway, which is known to be activated by damage caused by herbivores and wounds in tomatoes [118] and is a key factor in the development of plant defense mechanisms. This is how the damage caused by the nematode and the activation of this pathway are related.

Additionally, the glutaredoxin function was down-regulated at 2 dpi in the compatible but not in the incompatible interaction. As it was previously demonstrated in the incompatible interaction of tomato with *Phytophthora infestans*, enhanced expression of glutaredoxins reduces reactive oxygen species (ROS) accumulation and alleviates cell membrane injury, thus enhancing tomato resistance [119].

##### Late Phase of the Compatible Interaction (12 dpi)

The most overexpressed gene in this group, assigned to the Hormone Metabolism category, was related to induction and regulation of gibberellin and to a precursor of the antimicrobial peptide Snakin-1 (SN1) from potato. SN1 belongs to the Snakin/GASA family, because of their similarity with members of the GASA family from Arabidopsis (Gibberellic Acid Stimulated in Arabidopsis) [120] and the GAST family from tomato (Gibberellic Acid Stimulated Transcript) [121]. Homologous genes have been identified in other crop species [122]. Snakin/GASA proteins are involved in a diverse range of functions including hormonal crosstalk, development, and defense against biotic and abiotic stress [123,124].

Furthermore, in the late phase of the compatible interaction, an increase was observed in the expression of genes involved in cell wall degradation or modification, which other authors had previously linked to the gall formation and the reprogramming suffered by the root, also at late stages such as 7 dpi [43] or 12 dpi [48]. This overexpression is generally related to the degradation of the cell wall to provide energy to the nematode and to form the feeding site [43,48,125], which is consistent with the data from the present study.

The overexpression of a gene related to a Gamma-thionin can also be highlighted. Thionins are involved in plant defense against parasites, primarily increasing cell membrane permeability [126], which provokes several subsequent effects, such as membrane depolarization, an increase in Ca^2+^ and K^+^ ion permeability and the activation of some enzymes [127], strengthening the initial toxicity and leading to cell destruction [128]. Gamma-thionins bear strong similarity to defensins, another family of peptides that are components of the innate immune system in plants [129], and they are able to inhibit insect digestive proteins and interact with specific membrane components to trigger intracellular signaling cascades that hinder pathogen growth [130,131,132].

Other genes in the Stress category were up-regulated at the late phase of the compatible interaction, one of them encoding a wound-induced protein and the other gene associated with the TSI-1 protein included in the PR10 protein group. The PR10 protein interacts with stress signaling molecules and cross talk with different stress signal transduction pathways [133].

Another up-regulated gene was related to the synthesis of jasmonic acid (LEJA1) and, therefore, implicated in defense against pathogens. The same gene accounts for one of the basal differences between plants with and those without the *Mi-1* gene in the absence of infection.

Several transcripts related to RNA and associated with the regulation of transcription were up-regulated at the late phase of the compatible interaction but not in the incompatible one. Two of the genes encoded for the IAA3 and IAA8 proteins, which belong to the Aux/IAA family, whilst a third transcript was related to another myb-related transcription factor (THM16). Positive regulation had already been reported for genes encoding transcription factors in Arabidopsis during compatible interaction with cyst-forming nematodes [48]. Early feeding and whitefly oviposition on the leaves of susceptible tomato repressed the expression of several transcription factors, but two of these genes were overexpressed at 12 dpi in both the compatible and the incompatible interactions [57].

As for the down-regulated genes at 12 dpi, exclusively in the compatible interaction, most of them were related to proteins and transport, similar to what had been detected in a much more localized way in giant cells at 2 dpi [43], bearing in mind that the analysis in the present work was performed on a larger portion of the root and, therefore, a more global effect was analyzed.

The down-regulation of a couple of genes encoding a Dif10 extensin-like protein and another extensin class I protein is interesting, as extensins make up the cell wall and they are widely associated with defense responses against different root pathogens [111,112]. However, it is important to point out that, in the present study, the expression of other extensins was also down-regulated at 2 and 12 dpi during both the compatible and incompatible interactions.

Regarding the Transport category, two genes were down-regulated at 12 dpi, exclusively in the compatible interaction. The *LePT2* gene, encoding an inorganic phosphate transporter, is highly expressed in root and may play a significant role in acquiring the nutrient under natural conditions [134]. The production of another protein similar to an aquaporin was also reduced in the susceptible plants; tomato aquaporins have a role in ion homeostasis and are essential for plant resilience under salt stress [135].

Regarding the Stress category, it is worth noting the down-regulation of the *Mdip1* gene, which is involved in the synthesis of germin-like protein (GLP). GLPs play diversified roles in plant development and defense response [136,137].

In the category of TCA/Org. Transformation, another gene encoding succinate dehydrogenase (SDH) was exclusively down-regulated in Moneymaker. SDH plays an important role in ROS production, being a direct source of ROS in plant mitochondria and regulating plant development and stress responses [138].

Finally, other genes involved in the regulation of the jasmonate and ethylene pathways were down-regulated. However, it is important to note that another gene involved in the synthesis of jasmonic acid (LEJA1) was overexpressed at this same phase of the compatible interaction and also showed higher basal expression in uninfected Motelle than in Moneymarker. These up- and down-regulated genes seem to be involved in the complex process of the synthesis-degradation of jasmonic acid, as discussed above.

##### Comparison Between the Early and the Late Phase of the Compatible Interaction

When the reprogramming of tomato transcriptome due to nematode infection was analyzed in Moneymaker, comparing between the early (2 dpi) and the late (12 dpi) stages of the compatible interaction, many differences were detected. Although this result was expected because nematode infection involves physiological and morphological changes that evolve over time from the moment the nematode penetrates the root cells, settles there, and develops inside it [43,125,139,140], the notable differences observed were striking and interesting. In fact, both phases share only two overexpressed genes (corresponding to a wound-induced protein and a subtilisin-like protease) and no down-regulated genes. The common overexpression of the wound-induced protein suggests that the wound damage caused in the root cells by the initial invasion of the nematode continues over time, during at least 12 days post-infection. Nevertheless, it should be noted that the difference in the expression of this gene between infected and uninfected plants was reduced at the later stage of infection compared with the difference observed at 2 dpi. On the other hand, subtilisin-like protein SBT4A belongs to the SBT3/4 subfamily of tomato subtilases [141]. Initial evidence for the importance of plant subtilisin-like proteins in plant–pathogen interactions was reported in tomato [142]. The accumulation of subtilases in the plant extracellular matrix plays an important role during pathogenesis, and subtilases from tomato and Arabidopsis may be linked to pathogen recognition and the activation of signaling processes [143,144,145]. In the present study, the difference in the expression of SBT4A also decreased over time, although to a lesser extent than in the case of the wound-induced protein.

The fact that there are so few differential genes in common between the two phases of the compatible interaction is consistent with previous results by other authors [46], where only a minority of genes were common to the two phases (5 and 10 dpi) analyzed in that study, probably reflecting a progressive evolution of the susceptible plant in response to nematode parasitism. The substantial difference between the two phases in the data presented here would support this assumption, in addition to the fact that the number of differential genes (both up- and down-regulated) was much higher at 12 dpi than it was at 2 dpi. Another study [43] also observed a marked increase in the number of differentially expressed genes at 7 dpi compared to 3 dpi. The main differential genes specific to the compatible interaction at each stage of infection have been reviewed in detail earlier in this discussion.

#### 3.2.3. Transcriptome Reprograming Exclusive of the Incompatible Interaction

##### Early Phase of the Incompatible Interaction (2 dpi)

The highest overexpression at 2 dpi in the incompatible interaction but not in the compatible interaction corresponded to genes coding for a Kunitz family of trypsin and protease inhibitor proteins, which functions as an endopeptidase inhibitor, located in the apoplast (cell wall) and involved in plant defense/stress responses. The soybean Kunitz trypsin inhibitor (SKTI) was the first member of this family to be identified [146] and members of this trypsin inhibitor (STI) family are among the most versatile protease inhibitors reported, being able to interact with proteases belonging to different mechanistic classes [147].

Additionally, the *CEVI57* gene was up-regulated in the early phase of the incompatible interaction but not in the compatible one. CEVI57 is a type-2 protease inhibitor (PI-II) found naturally in tomato, part of the plant’s immune system to inhibit digestive proteases in insects and pathogens [148]. The *CEVI16* gene was also up-regulated at this time point in the incompatible interaction, although not as markedly as *CEVI57*. Cevi16 peroxidase is constitutively expressed in healthy tomato roots, and it was induced by viroid infection in the aerial tissues accompanied by the induction of resistance to subsequent pathogenic attacks [148]. The *CEVI16* gene has been given a potential role in pathogen defense, as it could be involved in cell wall hardening by the biosynthesis of cutin and/or suberin [148]. Similar to how these *CEVI* genes are activated by wounding or viroid infection, the results presented in this work suggest that production of these key defense proteins also increases following nematode infection in the roots of resistant tomato.

The up-regulation of another gene encoding a L-allo-threonine aldolase-related protein was also interesting, as threonine aldolases (TAs) catalyze the second pathway of glycine biosynthesis cleaving threonine to produce glycine and acetaldehyde [149]. TAs or their homologues have been isolated from various organisms including bacteria, fungi, mammals, yeasts, protozoa, insects and plants. With regard to plants, it was demonstrated that threonine aldolase plays a role in the nutritional quality of Arabidopsis seeds [150]; meanwhile, the pathway involving threonine is significantly altered in tomatoes under drought stress [151]. The results of the present study suggest that the overproduction of TAs could also play a role during the initial phase of the defense response of *Mi-1*-resistant tomato against root-knot nematodes.

In this early phase of the incompatible interaction, the overproduction of other enzymes such as carbonic anhydrase III (Ca3) and LOC544149 endochitinase was also observed. Carbonic anhydrases (CAs) are ubiquitous in nature including higher plants and they are part of defense mechanisms induced upon attack by various pathogens [152,153,154]. CAs in tobacco and Arabidopsis have been identified as SA-binding proteins with an antioxidant role during bacterial and viral infections [96,155]. The chloroplast CA of tobacco plays a role in HPR during the disease resistance to *Pseudomonas syringae* [155]. A pepper CA is a positive regulator of hypersensitive cell death in plants, and its overexpression in Arabidopsis mediates plant responses to biotrophic, hemi-biotrophic and necrotrophic pathogens [153]. A carbonic anhydrase of potato was very differently expressed in compatible vs. incompatible interactions with *Phytophthora infestans* [156]. The up-regulation of CA in that study was consistent with the findings of the present work, as it was observed from 48 h after pathogen inoculation but only in the incompatible interaction and not in the compatible one. At the same time, a gene encoding LOC544149 endochitinase was also overexpressed only in the incompatible interaction. This chitinase is a pathogenesis-related protein, specific to tomato plants, involved in various metabolic processes as it degrades chitin, a component of fungal cell walls, although it has also been linked to plant resistance to other pathogenic organisms including nematodes as chitin is the essential component of nematode eggshell and pharynx. In agreement with the present results, it was previously reported from other pathosystems that the induction of various classes of chitinases was greater and faster during the incompatible than during the compatible interactions [157,158,159,160].

The main functions suppressed in the early phase of the incompatible interaction, but not in the compatible, are related to RNA and hormone metabolism. The most down-regulated gene encoded the WRKY transcription factor LOC100191120. It is known that WRKYs are a family of transcription factors involved in multiple regulatory and developmental functions [161,162] and respond to both biotic and abiotic stress [102,162,163]. WRKYs have also been extensively studied in tomato: Some of these genes were previously found to be overexpressed during both the compatible and incompatible tomato–nematode interactions, while the expression of other WRKYs was induced only during the incompatible interaction, notably WRKY72 [52], although it should be noted that this particular WRKY was not included in the Affymetrix chip used in the present study. As previously discussed, different *WRKY* genes intervene in transcriptional reprogramming during plant immune responses to specific pests or pathogens, with individual WRKYs contributing subtly as components of a transcriptional network that includes feedback loops and feed-forward modules [102]. As an example, it was mentioned earlier in this discussion that another transcription factor (WRK10) was less expressed in uninfected Motelle than in uninfected Moneymaker; meanwhile, the opposite situation was observed for other WRKs, as they were more expressed in Motelle than in Moneymaker when uninfected plants were compared. Nevertheless, the observed down-regulation of a gene encoding LOC100191120 could indicate that there is partial repression of transcription only in the carrying-*Mi-1* plants, detectable two days after inoculation with the nematodes, and that this repression occurs mainly through a WRKY transcription factor. The WRKY found here may be an interesting candidate for further studies on its specific function in the early phase of incompatible interaction.

Regarding the down-regulation of genes related to hormone metabolism in the early phase of the incompatible interaction only, a gene stands out which is involved in the synthesis of the extensin-like protein Ext1. As previously mentioned, extensins are widely associated with defense responses in the root against pathogens, as these proteins are one of the elements that make up the cell wall [51,110,164]. Moreover, a lipoxygenase precursor was also down-regulated, which is involved in the synthesis-degradation process of jasmonic acid (JA). As already noted in this discussion, it is known that there is a differential regulation of the main plant hormones SA, JA and ET after nematode infection [65,74,75]. The coordination and contribution of hormones in the specific case of tomato defense against nematodes is closely related to the *Mi-1* gene [65]. In the case of JA, it has been proven that this hormone is not essential in the *Mi-1*-mediated resistance of tomato, but its absence reduces susceptibility to nematodes in the compatible interaction [65]. The complexity of the role played by hormones is well known but, according to the present results, the metabolism of JA appears to be reduced at 2 dpi in the resistant tomato.

Several other genes were down-regulated in the early phase of the incompatible interaction, some encoding a pathogenesis-related protein (PR-P). Although the infection in both interactions is known to activate PRs [46], certain genes encoding proteins involved in signaling are suppressed by the nematode [91]. It was also demonstrated that most of the genes repressed by nematodes are stress-related, for example, in Arabidopsis and tomato galls [43]. Despite some advances in the identification of possible defense genes that nematodes can suppress in the plant when they invade it [165,166], the mechanism by which this suppression occurs remains largely unknown.

##### Late Phase of the Incompatible Interaction (12 dpi)

The most overexpressed gene in the late phase of the incompatible interaction, but not in the compatible one, encoded the enzyme hyoscyamine 6-dioxygenase (H6H), which directly catalyzes the formation of scopolamine in the tropane alkaloids biosynthesis pathway, often localized in roots [167]. Interestingly, the expression of the same gene was slightly reduced in the early phase of the compatible interaction. Scopolamine is a defense compound in Solanaceae plants that protects them from herbivores and pathogens by acting as a potent anticholinergic. For some time now, H6H has been considered a promising enzyme to enhance the yields of scopolamine in plant roots by means of metabolic and genetic engineering [168].

Similarly, another gene encoding a metallocarboxypeptidase inhibitor was also overexpressed in Motelle at 12 dpi, which had appeared slightly down-regulated in the early phase of the compatible interaction. Many of these inhibitors are active against proteases from the digestive tracts of insects, and their expression in plants is activated by mechanical wounding or insect injury. They are, thus, thought to be part of a plant’s defense system against insect attack [169,170] and, now, also for the resistance to infection by *M. javanica*.

Also interesting is the up-regulation of a gene with a defense function in synthesizing AAD50436, a hypersensitive response-assisting protein (HRAP). According to a previous study on the resistance conferred by the RB gene of potato against *Phytophthora infestans*, the same protein AAD50436 was expressed at a higher level in resistant potato lines than in the susceptible control after 72 h post infection [171].

Several other proteins involved in defense against pathogens were notably overexpressed at 12 dpi, only in the incompatible interaction. Among them, the most overexpressed was chalcone synthase (CHS) B. Chalcones defend the plant against pathogens, as they are precursors for anthocyanins and flavonols, which regulate plant pigmentation and are required for plant protection and response to biotic and abiotic stresses [172]. Additionally, a gene was up-regulated that encodes lipoxygenase A, which is involved in JA biosynthesis and has been reported to be induced during RKN infection in tomato, among other plants [173]. Similarly, it is worth mentioning the up-regulation of the pathogenesis-related protein leaf 6 (P6) and the pathogenesis-related protein 5 (PR-5x), both involved in the plant response to pathogens [174].

Overexpression of an ornithine decarboxylase of tomato (LEODC) together with the SPM1 protein is interesting, as ODC in plants catalyzes the first step of the polyamines (PAs) biosynthesis that converts ornithine into putrescine, which is the precursor for spermidine and spermine; meanwhile, SPM1 is involved in the subpathway that synthesizes spermidine from putrescine [175]. During plant–pathogen interactions, plants use specific PAs to enhance their immunity; meanwhile, pathogens use certain PAs to facilitate the plant invasion [176].

A gene encoding snakin-2 (SN2) peptide was also up-regulated at the late stage of the incompatible interaction. SN2 is related to abiotic stress as part of the jasmonate-dependent signaling pathway, and exhibits strong antimicrobial activity against bacteria and fungi [177]. Moreover, SN2 levels increased in tomato upon wounding, fungal infection or application of methyl jasmonate [178]. The expression of another member of the snakin family (CaSnakin) was induced by root-knot nematode infection in a resistant pepper cultivar, and the *CaSn* gene plays an important role in host defense against nematodes [179].

Another gene up-regulated at the late phase of the incompatible interaction was the *fer* gene, which encodes a protein containing a basic helix–loop–helix domain (bHLH transcription factor). This tomato gene is expressed in a cell-specific pattern at the root tip independently from iron supply and it was the first identified regulator for iron nutrition in plants [180]. Moreover, *fer* gene expression was down-regulated at a high or sufficient iron supply compared to a low supply [181]. In the present study, the same gene was also up-regulated in the early phase of the incompatible interaction, although with a slightly lower FC value. Although it was not possible to establish a direct link between the *fer* gene and the resistant plant response to pathogens, it is known that several bHLH transcription factors are involved in the JA response network and one of them might perform some function in the TYLCV infection of tomato [182].

The gene encoding S-Adenosyl-L-Methionine: Salicylic Acid Carboxyl Methyltransferase (SAMT) enzyme, which converts salicylic acid (SA) to methylsalicylate, was also up-regulated at this time point of the tomato resistance to *M. javanica*. SA pathways are crucial for plant immunity, and SAMT’s activity contributes to both local and systemic defense responses to pathogens [183,184]. It was demonstrated that tomato SAMT is critical for methyl salicylate synthesis and methyl salicylate, in turn, likely has an important role in controlling SA synthesis [185]. Overexpression of a gene encoding SAMT in the hairy root system of soybean could confer resistance to the soybean cyst nematode *Heterodera glycines* [186]. Moreover, SAMT is a component of a signaling circuit mediating airborne defense against aphids and viruses [187].

Also interesting is the up-regulation of a gene related to the Rubisco small subunit precursor (RbCS) of *Nicotiana benthamiana*, which plays a vital role in tobamovirus movement and plant antiviral defenses [188].

A gene encoding the CAA06997 subtilisin-like protease was up-regulated in the late phase of the incompatible interaction but not in the compatible interaction, although a different subtilisin-like protein (SBT4A) had been previously up-regulated in both the early and the late phases of the compatible interaction. As stated above, the importance of plant subtilisin-like proteins in plant–pathogen interactions was first reported in tomato [142], and subtilases from tomato and Arabidopsis have been linked to pathogen recognition and activation of signaling processes [143,144,145].

Finally, it was unexpected that a gene encoding expansin9 (EXPA9) protein was up-regulated in the late phase of the incompatible interaction (but not in the compatible), as EXPA9 causes the loosening and extension of plant cell walls. Previous studies demonstrated that the expression of several expansins was up-regulated during the development of giant cells induced by root-knot nematodes in susceptible plants [45,87]. Interestingly, another 3 different expansins were found, in the present work, to be less expressed in the resistant cultivar Motelle than in Moneymaker, both in the absence of infestation, which would contribute to impairing in Motelle the compatible interaction that occurs in Moneymaker. Taken together, these data seem to indicate that not all expansins exhibit the same dynamics in their expression following nematode infection.

Regarding the genes down-regulated at 12 dpi in the incompatible interaction, the most repressed compared to uninfected plants was the tomato *ACO5*, which is the most divergent among *ACO* gene family members [87]. *ACO* has been isolated and characterized in many plant species including tomato [189] and encodes for 1-aminocyclopropane-1-carboxylate oxidase, which converts 1-aminocyclopropane-1-carboxylic acid (ACC) to ethylene (ET). Moreover, the expression of several ethylene responsive element binding (EREB) proteins was reduced at the same time. Down-regulation of ACO5 enzyme production, together with the decrease in the amount of these EREB proteins in the late stage of the incompatible interaction Motelle/*M. javanica* leads to reduced ET production, probably due to the activation of the salicylic acid (SA) pathway, which is the main pathway involved in the signaling that leads to the *Mi-1*-mediated resistance of tomato to root-knot nematodes [190]. It is known that the SA and JA/ET signaling pathways are antagonistic and communicate mainly by negative cross-talk [47]. In agreement with all of this, it is important to mention the down-regulation, at the same time point, of a lipoxygenase precursor that is involved in the synthesis-degradation process of jasmonic acid (JA), and that it had already appeared down-regulated in the early phase as well. Probably related to the reduced activity of the JA/ET signaling pathway in the resistance to the nematode, a gene encoding the copalyl diphosphate synthase (CPS) was notably down-regulated, being CPS one of the few diterpene synthases characterized in tomato. Some diterpene synthases are induced by MeJA in different plant species [191], so reducing the availability of MeJA would lead to a decrease in the amount of CPS enzyme.

In addition to the aforementioned phytohormones, auxin plays a significant role in regulating plant growth and development but also in mediating stress responses to pathogens in a number of plant species. Interestingly, two genes down-regulated in the late phase of the incompatible interaction are auxin early response genes (AERGs), involved in the auxin perception and regulation of the signal transduction pathway [192]. IAA2 protein is a transcription factor that functions as a repressor of early auxin response genes at low auxin concentrations, with *Aux/IAA* genes implicated in nearly every aspect of plant growth and developmental processes, including adjustment in biotic stress conditions [193]. Members of the *SAUR* gene family are implicated in the IAA signaling response pathway [194]. Up- and down-regulation of these and other genes involved in auxin modulation are part of the complex signaling network between auxin and other phytohormones to mitigate plant biotic and abiotic stresses [195].

A gene encoding a lipid transfer protein (LTP) was notably down-regulated at the late phase of the incompatible interaction but not in the compatible one. LTPs bind and transfer lipids and are components of plant innate immunity. Moreover, they constitute one of the classes of defense PRPs, many of which have antimicrobial and enzymatic activities or are enzyme inhibitors [196].

Also down-regulated was the expression of the receptor-like protein EIX1, which is included in RLP family with biotic stress function as it is a potent elicitor of plant defense responses in tobacco and tomato [197].

A slight down-regulation of the gene encoding mitogen-activated protein kinase 3 (MPK3) was unexpected in the incompatible interaction of the present study, as MAPKs in tomato and other plant species are implicated in the resistance against root-knot nematodes [197,198]. However, information on the interaction of phytohormones with reactive oxygen species (ROS) signaling and MAPK cascades in nematode resistance is scarce [198]. Therefore, the occasional and not-very-pronounced reduction in MPK3 levels could be the result of the activation/deactivation of other genes in this complex signaling network.

##### Comparison Between the Early and the Late Phase of the Incompatible Interaction

The number of differential transcripts between infected and uninfected Motelle plants was far higher at 12 dpi than at 2 dpi, even more so than in the case of the compatible interaction. Moreover, the genes or transcripts common to both phases of the incompatible interaction were scarce: only one up-regulated gene and two down-regulated.

The gene encoding a methionine gamma-lyase (MGL) was up-regulated in both phases of the incompatible interaction, with a slight increase in differential expression over time. Interestingly, the same gene had been also detected at 2 dpi in the compatible interaction but not later. MGL catabolizes methionine, leading to the synthesis of isoleucine (Ile) [199] and overexpression of this gene in Arabidopsis conferred resistance to the beet cyst nematode *Heterodera sacchari* [200]. By paying attention to the temporal evolution of FC values in each of the interactions, it can be deduced that, in both interactions, the activity of this gene increased at 2 dpi compared to uninfected plants, but slightly more in Motelle than in Moneymaker. However, 10 days later, gene activity in the infected susceptible plants had decreased slightly so that differential expression with uninfected plants is no longer detected by microarray analysis. Conversely, it continued to increase in nematode-resistant plants until the difference with uninfected plants became even more evident. Therefore, it appears that the activity of this gene may be related to resistance to *M. javanica* mediated by the *Mi-1* gene.

The gene encoding ABA 8’ hydroxylase CYP707A1 was slightly and similarly down-regulated in both phases of the incompatible interaction, although it had been up-regulated at 2 dpi in the compatible interaction. Abscisic acid (ABA) is a phytohormone for balancing plant growth and adaptation to biotic and abiotic stresses [201]. In a pathogen infection, ABA modulates the JA and SA pathways to trigger the plant response. Then, if environmental conditions improve, ABA levels decrease, allowing growth hormones such as IAA to regain their dominance [201]. The hydroxylation at the 8′-position of ABA is known as the key step of ABA catabolism, and this reaction is catalyzed by ABA 8′-hydroxylase CYP707As [202]. Therefore, the down-regulation of the gene encoding ABA 8’ hydroxylase CYP707A1, sustained over time throughout the incompatible interaction, would lead to maintaining high levels of ABA, which would contribute to ensuring the plant’s resistant response to the nematode.

Finally, a lipoxygenase gene (*loxD*) is noteworthy, which also appeared down-regulated upon infection by *M. javanica* in Motelle, with slightly higher FC values in the early than in the late phase. This result is consistent with those obtained by other authors [203], who cloned and characterized in pea a lipoxygenase gene (*LOXN2*) related to defense against the cyst nematode *Heterodera goettingiana*. Following nematode infection, expression of this gene was down-regulated in the resistant (but not in the susceptible) pea genotype, thus showing a trend opposite to that of other *LOX* genes. The results of the present study confirm that *LOX* family members respond differentially to the infection, with some *LOX*s, together with other defense genes, decreasing their expression during pathogen infection [204].

As explained for the compatible interaction, the main differentially expressed genes specific to the incompatible interaction at each of its temporal stages have already been revised in detail in other sections of this discussion.

## 4. Materials and Methods

### 4.1. Plant Material and Growth Conditions

Tomato (*Solanum lycopersicum* L.) plants of two near-isogenic lines were used, RKN-resistant Motelle (*Mi-1*/*Mi-1*) and RKN-susceptible Moneymaker (*mi-1*/*mi-1*), which differ only by the presence of a 650 kb introgressed region from *Solanum peruvianum* containing the *Mi-1* gene in Motelle cultivar [63,205].

Seeds were surface sterilized with 10% bleach for 5 min and sown in seedling trays filled with autoclaved vermiculite (number 3, Projar, Valencia, Spain). Tomato seeds were allowed to germinate in a growth chamber at 25:20 °C, 16:8 (L:O) and 70% R.H. Four weeks after germination, plants were transplanted to 15 cm diameter plastic pots filled with an organic planting mix (45% river sand, 45% “miga sand” and 10% organic matter) and maintained under the same conditions. Plants were fertilized once every other week with a 3 gr·L^−1^ solution of the nutritive complex 20-20-20 (Nutrichem^®^ 60; Miller Chemical, Hanover, PA, USA) and irrigated with tap water when needed in the meantime. Eight-week-old plants were used for the assays.

### 4.2. Nematodes and Root Infections

Root-knot nematodes (*M. javanica* (Treub) Chitwood) were obtained from a culture on susceptible tomato cv. Marmande maintained in a growth chamber under the above detailed climatic conditions. Nematode eggs were collected by chopping the infected roots and treating them with a 10% sodium hypochlorite solution. The resulting liquid was poured through two sieves with 0.07 and 0.025 mm pores. The retained eggs were allowed to hatch for 48–72 h at room temperature inside a Petri dish, on a wet filter paper in contact with a small volume of water.

When tomato plants were eight weeks old, at least 20 plants from each cultivar were inoculated with 3000 freshly hatched juvenile nematodes (J2) per plant. Other 20 plants per cultivar were not inoculated and used as uninfected control plants.

### 4.3. Sampling of Root Tissues

Samples of tomato roots were taken at two moments after nematode inoculation: 2 and 12 days post infection (2 dpi and 12 dpi, respectively).

At each sampling time, the whole root systems of 3 infected plants from the same tomato genotype were collected, gently washed and mixed. From this mix, a 3 grs biological sample was obtained, immediately frozen in liquid nitrogen and stored at −80 °C until RNA extraction. Similarly, uninfected plants were separately processed at the same time. Three biological samples from each genotype (Moneymaker or Motelle) and treatment (infected or uninfected) were collected.

### 4.4. RNA Isolation and Microarray Hybridization

Each frozen root sample was ground in liquid nitrogen using a mortar and RNA extraction was performed using Trizol^®^ Reagent (Thermo Fisher Scientific, Waltham, MA, USA). The integrity of the extracted RNA was determined using a Bioanalyzer 2100 (Agilent Technologies, Santa Clara, CA, USA).

The GeneChip^®^ Tomato Genome Array of Affymetrix^®^ (Santa Clara, CA, USA) was used for the microarray hybridization. This chip, especially designed to analyze tomato expression, is obtained by synthesizing oligonucleotides of 25 bases, using the photolithography technique on a quartz surface (http://www.affymetrix.com/products_services/arrays/specific/tomato.affx) accessed on 15 October 2014. RNA samples were labeled with biotin and each sample was hybridized to a separate array containing over 10,000 *S. lycopersicum* probe sets to monitor the expression of over 9200 transcripts and allow the identification of those having a differential expression between two compared treatments. cDNA synthesis and cRNA production and fragmentation for microarray hybridization were performed as described in the Expression Analysis Technical Manual (Affymetrix^®^, Santa Clara, CA, USA). The Platform GeneChip^®^ Scanner 3000 7G System (Affymetrix^®^, Santa Clara, CA, USA) including the Fluids Station model 450 as well as the GeneChip^®^ Scanner model “3000 7G” for image catching were used for RNA processing.

### 4.5. Data Analysis

The data obtained from the microarrays were analyzed using *limma* [206] applying the Robust Multiarray Average (RMA) [207] to adjust the background, normalize, and log-transform values. Raw *p* values were adjusted for multiple hypotheses testing using the false discovery rate (FDR) method [208]. Differentially expressed transcripts were identified on the basis of an adjusted *p* value < 0.05 for FDR. Changes of at least twice or half in expression were selected by establishing the fold change values as ≥2 and ≤−2 for up- and down-regulated transcripts, respectively.

The VENNY software version 2.1 [209] was used to identify common genes between two lists of selected transcripts corresponding to different experimental situations.

Descriptions of the genes and target sequences corresponding to GeneChip probesets were obtained from Affymetrix, Tomato Annotations Release 36 (NetAffx Analysis Center). Target sequences were also used in BLAST (version 2.2.27) searches of their corresponding tomato genes (version SL3.0 and Annotation ITAG4.1) in Sol Genomics database [210].

The functional classification of the differentially expressed genes was carried out using the MAPMAN software version 3.1.0 (http://mapman.gabipd.org/web/guest/mapman) (accessed on 20 May 2015). Analysis of GO term enrichment was performed with the Singular Enrichment Analysis (SEA) tool in AgriGO V2.0 [211]. Significant terms (*p*-value < 0.05, Fisher’s exact test) were selected for plotting with the ‘ggplot2’ R library [212].

### 4.6. Validation of Microarray Data by qRT-PCR

The transcripts used for the qRT-PCR and the selected interactions and time points are listed in Table 4. These transcripts were previously verified in the “Tomato Functional Genomics Database” (http://ted.bti.cornell.edu/) (accessed on 20 May 2015) and SGN (Sol Genomics Network, http://solgenomics.net/) (accessed on 20 May 2015) that corresponded to genes with full sequence data, in order to design specific qPCR primers. The primers were designed using Primer-Blast programme (http://www.ncbi.nlm.nih.gov/tools/primer-blast/) (accessed on 20 May 2015) using only those that would take the UTR3 to ensure they were highly specific, and they amplified a region no longer than 200 bps. Sequences of the primers are provided as Supplemental Data (Table S2).

For qRT-PCR, 2 μg of total RNA was used, following the instructions for the High-Capacity cDNA Reverse Transcription Kit (Applied Biosystems ^®^, Foster City, CA, USA) in the presence of an oligo-dT oligonucleotide and in a final volume of 20 μL. Quantitative PCRs were performed on Applied Biosystems^®^ ViiA™ 7 384-well plates using SYBR^®^ Green (Applied Biosystems^®^, Foster City, CA, USA). Three technical replicates were performed for two biological samples of each gene, and relative expression (RE) was determined with respect to the endogenous 18S rRNA amplification with Eukaryotic 18S rRNA Endogenous Control FAM™/MGB probe, non-primer limited (Applied Biosystems^®^, Foster City, CA, USA).

RE data obtained by both qRT-PCR and microarray were transformed to a logarithmic scale Log_2_(x), and the Pearson correlation coefficient (r) was calculated using the GraphPad Prism program (version 11.0.0 for Windows, GraphPad Software, LLC, San Diego, CA, USA, www.graphpad.com). The program also calculates the *p*-value to establish whether the correlation between both variables is statistically significant.

## 5. Conclusions

Comparing transcriptomic profiles of Moneymaker and Motelle roots reveals baseline differences even without infection, likely linked to the *Mi-1* gene in Motelle. Motelle shows higher expression of 180 transcripts (including those corresponding to *Mi-1*) and lower expression of only 44, with higher levels of RE in the up- than in the down-regulated transcripts. Genes related to salicylic, jasmonic, and ethylene pathways are more active in Motelle, suggesting enhanced hormonal activity. Conversely, the Gibberellic acid insensitive mutant protein (GAI) is the most repressed in Motelle compared to Moneymaker.

Infection by *M. javanica* triggers strong transcriptional reprogramming in both Moneymaker and Motelle roots, increasing over time. At the early stage of infection (2 dpi), slightly more genes are up- or down-regulated in the compatible interaction than in the incompatible one, whereas at the late stage (12 dpi), the opposite pattern is observed. At both time points, few differential genes are shared between both interactions, and most of them are up-regulated.

At 2 dpi, genes uniquely up-regulated in the compatible interaction are related to the cell wall, hormone metabolism, RNA, stress, and transport, while genes linked to signaling, protein, and redox are down-regulated. Genes encoding a family of trypsin and protease inhibitor proteins are up-regulated exclusively in the incompatible interaction, whereas genes related to hormone metabolism and RNA are down-regulated.

At 12 dpi, main genes uniquely up-regulated in the compatible interaction are linked to hormone metabolism, the cell wall, RNA, stress, defense, and development, while genes related to transport, hormone metabolism, or encoding extensin and other defense or stress proteins are down-regulated. In the incompatible interaction, genes involved in hormone metabolism, stress, and pathogen defense are up-regulated, while others in the same functional categories are down-regulated, in an activation/deactivation process that modulates the complex network of phytohormone pathways involved in plant defense.

## Figures and Tables

**Table 1:** Transcripts up-regulated in tomato roots by *M. javanica* at 2 dpi, common to both compatible and incompatible interactions.

Affymetrix ID ^1^	Mapman Function ^2^	TFGD Function ^3^	Relative Expression (Fold Change) ^4^	
			Compatible	Incompatible
Les.5916.1.S1_at	RNA. Regulation of transcription. bZIP transcription factor family	Anaerobic basic leucine zipper protein (ABZ1) [*Solanum lycopersicum*]	3.14	2.72
Les.2001.1.S1_s_at	RNA. Regulation of transcription. Unclassified	Hypothetical protein [*Solanum lycopersicum*]	2.64	3.88
LesAffx.69992.1.S1_at	Amino acid metabolism. Synthesis. Aspartate family. Methionine	Predicted protein [*Populus trichocarpa*]	2.03	2.16

^1^ Affymetrix ID is the identifier of each transcript according to Affymetrix database. ^2^ Functional description of transcripts according to Mapman Program. ^3^ TFGD Function indicates functional description of transcript according to Tomato Functional Genomics Database. ^4^ Fold change represents the expression value obtained at two days after nematode inoculation, by comparing infected with uninfected plants of the same tomato cultivar: Moneymaker (compatible interaction) or Motelle (incompatible interaction).

**Table 2:** Transcripts up- or down-regulated in tomato roots by *M. javanica* at 12 dpi, common to both compatible and incompatible interactions.

Affymetrix ID ^1^	Mapman Function ^2^	TFGD Function ^3^	Relative Expression (Fold Change) ^4^	
			Compatible	Incompatible
Les.5442.1.S1_at	RNA.regulation of transcription.Aux/IAA family	Predicted protein [*Populus trichocarpa*]	4.70	3.05
LesAffx.30937.1.S1_at	Misc.cytochrome P450	Unnamed protein product [*Vitis vinifera*]	4.22	5.52
LesAffx.26129.1.S1_at	Not assigned.unknown	Predicted: hypothetical protein [*Vitis vinifera*]	3.77	2.26
Les.4596.1.S1_at	Not assigned.unknown	Defensin protein [*Solanum pimpinellifolium*]	3.46	5.27
Les.4300.1.A1_at	Not assigned.unknown	No hits found	3.30	2.93
LesAffx.9038.1.S1_at	Misc.cytochrome P450	Cytochrome P450 monooxygenase [*Petunia × hybrida*]	3.11	2.13
Les.4333.1.S1_at	Lipid metabolism.lipid transfer proteins etc	RecName: Full = Non-specific lipid-transfer protein 1; Short = LTP 1; Flags: Precursor	2.99	3.49
LesAffx.60225.1.S1_at	Hormone metabolism.abscisic acid.induced-regulated-responsive-activated	HVA22-like protein c, putative [*Solanum demissum*]	2.85	2.83
Les.4938.1.S1_at	Not assigned.unknown	Unknown [*Populus trichocarpa*]	2.63	3.96
Les.1851.1.A1_at	Development.unspecified	No hits found	2.61	2.04
Les.3055.1.S1_at	Cell.cycle	CycD3;3 [*Solanum lycopersicum*]	2.54	2.16
Les.41.1.S1_at	RNA.regulation of transcription.MYB domain transcription factor family	Transcription factor [*Solanum lycopersicum*]	2.38	2.52
LesAffx.68802.1.S1_at	Misc.short chain dehydrogenase/reductase (SDR)	Predicted: hypothetical protein [*Vitis vinifera*]	2.18	2.24
Les.83.1.S1_at	RNA.regulation of transcription.HB, Homeobox transcription factor family	RecName: Full = Homeotic protein knotted-1; Short = TKN1	2.17	2.95
Les.2504.1.A1_at	Hormone metabolism.salicylic acid.synthesis-degradation	No hits found	2.16	4.98
Les.2700.1.S1_at	Not assigned.disagreeing hits	No hits found	2.16	2.05
Les.1389.1.S1_at	Lipid metabolism.lipid transfer proteins etc.	RecName: Full = Non-specific lipid-transfer protein 2; Short = LTP 2; Flags: Precursor	2.11	2.10
LesAffx.60152.1.S1_at	RNA.regulation of transcription.HB, Homeobox transcription factor family	WOX4 [*Solanum lycopersicum*]	2.07	2.63
Les.5112.1.S1_at	Transport.Major Intrinsic Proteins.PIP	Silicon transporter, putative [*Ricinus communis*]	−2.02	−3.34
LesAffx.64062.1.S1_at	Stress.abiotic.unspecified	Germin like protein [*Nicotiana tabacum*]	−2.08	−3.49
Les.228.1.S1_at	Misc.protease inhibitor/seed storage/lipid transfer protein (LTP) family protein	Putative proline-rich protein [*Lycopersicon esculentum*]	−2.15	−5.68
Les.4149.3.S1_at	Signalling.calcium	39 kDa EF-Hand containing protein [*Solanum tuberosum*]	−2.61	−2.75
LesAffx.21576.1.S1_at	Misc.protease inhibitor/seed storage/lipid transfer protein (LTP) family protein	Extensin-like protein [*Capsicum chinense*]	−2.72	−4.04
Les.228.1.S1_a_at	Misc.protease inhibitor/seed storage/lipid transfer protein (LTP) family protein	Putative proline-rich protein [*Lycopersicon esculentum*]	−3.13	−6.38

^1^ Affymetrix ID is the identifier of each transcript according to Affymetrix database. ^2^ Functional description of transcripts according to Mapman Program. ^3^ TFGD Function indicates functional description of transcript according to Tomato Functional Genomics Database. ^4^ Fold change represents the expression value obtained at 12 days after nematode inoculation, by comparing infected with uninfected plants of the same tomato cultivar: Moneymaker (compatible interaction) or Motelle (incompatible interaction).

**Table 3:** Transcripts differentially expressed in tomato roots by *M. javanica* infection, common to 2 and 12 days post-inoculation.

Affymetrix ID ^1^	Mapman Function ^2^	TFGD Function ^3^	Relative Expression (Fold Change) ^4^	
			2 dpi	12 dpi
Compatible interaction:				
Les.2476.1.S1_at	Stress. Abiotic. Touch/Wounding	CAA42537 wound induced protein [*Solanum lycopersicum*]	4.02	3.17
LesAffx.8850.1.S1_at	Not assigned. No ontology	AAF77578 pepper esterase [*Capsicum annuum*]	2.28	2.19
Incompatible interaction:				
LesAffx.69992.1.S1_at	Amino acid metabolism. Synthesis. Aspartate family. Methionine	XP_002299428 predicted protein [*Populus trichocarpa*]	2.16	3.27
LesAffx.3253.2.S1_at	Miscelaneous. Cytochrome P450	ABA55732 ABA 8′-hydroxylase CYP707A1 [*Solanum tuberosum*]	−2.02	−2.01
Les.3632.1.S1_at	Hormone metabolism. Jasmonate. Synthesis-degradation. Lipoxygenase	AAB65767 lipoxygenase	−2.38	−2.07

^1^ Affymetrix ID is the identifier of each transcript according to Affymetrix database. ^2^ Functional description of transcripts according to Mapman Program. ^3^ TFGD Function indicates functional description of transcript according to TFGD database (Tomato Functional Genomics Database). ^4^ Fold change represents the expression value of transcript at 2 and 12 days post-inoculation. Data from compatible and incompatible interactions are presented separately.

**Table 4:** Transcripts used for validation of microarray analysis by means of qRT-PCR.

Affymetrix ID	Interaction and Time Point	SGN ID ^1^	Relative Expression ^2^ (Log_2_ RE)	
			Microarray	qRT-PCR
Les.3974.1.A1_at	Incompatible 12 dpi	SGN-U579158	2.29	1.64
Les.3809.2.S1_a_at	Compatible 2 dpi	SGN-U565616	1.95	1.74
Les.2476.1.S1_at	Compatible 12 dpi	SGN-U579880	1.66	1.13
Les.100.1.S1_at	Compatible 12 dpi	SGN-U594480	−2.05	−0.4
Les.3632.1.S1_at	Incompatible 2 dpi	SGN-U569257	−1.25	−0.59
Les.3632.1.S1_at	Incompatible 12 dpi	SGN-U569257	−1.05	−0.14

^1^ SGN ID is the identification number corresponding to each transcript according to Sol Genomics Network. ^2^ The relative expression (RE) values were transformed by means of Log_2_(X) previous to calculate the Pearson Correlation coefficient.

## Data Availability

The original contributions presented in this study are included in the article/supplementary material. Further inquiries can be directed to the corresponding author.

## Supplementary Materials

The following supporting information can be downloaded at: https://www.mdpi.com/article/10.3390/plants15101428/s1, Table S1: Lists of genes differentially expressed between Moneymaker and Motelle and during the tomato/*M. javanica* interactions. Table S2: Primers used for qRT-PCR to validate results from microarray analysis.
