# Supplementary material for: Basal Differences in the Transcriptional Profiles of Tomato Roots Associated with the Presence/Absence of the Resistance Gene Mi-1 and Time-Course Changes During the Compatible and Incompatible Interactions with the Root-Knot Nematode Meloidogyne javanica
Source: Plants (Basel). 2026 May 7;15(10):1428. doi: 10.3390/plants15101428 (PMC13210716; doi:10.3390/plants15101428)
Supplement: Supplementary file 1 [file plants-15-01428-s001.zip › Supplemental Table S2.pdf]

Table S2. Primers used for qRT-PCR to validate results from microarray analysis.

SGN ID	Affymetrix ID	Primers	Sequences
**SGN-U579158**	Les.3974.1.A1_at	158 F<br>158 R	5′-TGTTCTGGTGGTACGTTCTGT-′3<br>5′-ACGAAATTGTAATCACACGCCT-′3
**SGN-U565616**	Les.3809.2.S1_a_at	2d 616 F<br>2d 616 R	5′-ACGTACGTGTACAAGGAGAAGA-′3<br>5′-AGTGACAGAGTGTGATCCTGT-′3
**SGN-U579880**	Les.2476.1.S1_at	2d 880 F<br>2d 880 R	5′-TGAGCTGTTGGGGTCCAAAT-′3<br>5′-ACACAATGTATTGCAGTTTTCTGA-′3
**SGN-U594480**	Les.100.1.S1_at	4480 F<br>4480 R	5′-AAGTCCCCGTCCCCTTCAA-′3<br>5′-GGAGACTTGTAGTAAACAGGCG-′3
**SGN-U569257**	Les.3632.1.S1_at	2 y 12 D 257 F<br>2 y 12 D 257 R	5′-AGGTGTGGTGCTGGTGTATT-′3<br>5′-CACAGCAGTCCGCCCTATTT-′3

[1] S

GN ID is the identification number corresponding to each transcript according to Sol Genomics Network. [2] The relative expression (RE) values were transformed by means of $Log_2(X)$ previous to calculate the Pearson Correlation coefficient.
